# Optimizing ATP Isothermal Tests: A Theoretical and Experimental Approach

**DOI:** 10.3390/e28010047

**Published:** 2025-12-30

**Authors:** Juan P. Martínez-Val Piera, Alberto Ramos Millán

**Affiliations:** 1 Túnel de Frío, Fundación para el Fomento de la Innovación Industrial, Eric Kandel, 1, 28906 Getafe, Madrid, Spain; juan.mpiera@gmail.com; 2 Department of Energy and Fuels, Universidad Politécnica de Madrid, Alenza, 4, 28003 Madrid, Spain

**Keywords:** optimization, transportation, controlled temperature, isothermal tests, ATP, energy optimization

## Abstract

The International Agreement on the Carriage of Perishable Foodstuffs and on the Special Equipment to Be Used for Such Carriage (usually known as ATP Treaty) defines a standardized isothermal test for qualifying refrigerated containers, but its current protocol is lengthy, costly and lacks scientific justification. This paper presents a combined theoretical and experimental study aimed at optimizing this procedure. First, a heat-transfer framework based on transient conduction and thermal diffusivity is developed to estimate stabilization times using dimensionless criteria. Then, extensive experimental tests on ATP containers validate these predictions and reveal additional phenomena such as air leakage and chimney effects. Based on these findings, a revised protocol is proposed that reduces the test duration from more than 18 h to approximately 2 h while preserving the thermal stabilization conditions required by ATP. Experimental results show that the uncertainty in the determination of the global heat-transfer coefficient *K* is reduced from about 2–2.3% in the classical ATP procedure to roughly 0.7–1.0% with the new protocol. In addition, the method suppresses secondary physical effects—such as chimney-driven air leakage and latent-heat losses due to water evaporation—thus improving the physical representativeness of the measured *K* value. The proposed accelerated protocol offers a scientifically grounded, cost-effective alternative for future ATP standards.

## 1. Introduction

Most of the current social and economic activities critically depend on the supply of technical services. For instance, a general blackout in the electric grid would totally destroy our way of living. On a much smaller scale, failure of our cooling and freezing devices can pose a severe problem for foodstuff conservation. This fact was clearly known 50 years ago when the International Agreement on the Carriage of Perishable Foodstuffs and on the Special Equipment to Be Used for Such Carriage (usually known as ATP Treaty) was agreed upon and signed for the first time in Geneva, Switzerland, in 1970 [1]. The General Secretariat of the Treaty officially is the UN Secretary-General, although the administration and day-to-day secretariat was conferred to the United Nations Economic Committee for Europe (UNECE), particularly its Working Party 11. It must be noted that the Treaty includes countries from other continents and is becoming larger [2].

The ATP Agreement defines standardized procedures to certify the thermal performance of insulated transport equipment used for carrying perishable foodstuffs. In practice, the key performance indicator is the global heat-transfer coefficient *K*, obtained from an isothermal test in which the container is subjected to imposed temperature differences between its interior and the surrounding test hall. The current protocol, however, requires long testing times (typically more than one day including preparation and steady-state phases) and provides a single steady-state measurement, with no explicit assessment of its statistical significance or the influence of secondary physical effects such as air leakage or wall moisture.

The thermal behavior analyzed in this work is governed by irreversible heat-transfer processes and transient diffusion, which fall within the scope of Entropythrough the study of transport phenomena, stability and non-equilibrium thermodynamics. Although the manuscript does not present explicit exergy or entropy balances, it addresses the fundamental mechanisms controlling entropy-producing thermal diffusion in insulated systems, and proposes a revised protocol grounded on these physical principles.

The Treaty mainly was a political agreement, and it was conceived to include all types of possibilities in the different markets of the involved countries. Such a political root includes a condition in the Treaty that makes it very difficult to change a single word in it, namely, all variations must be approved unanimously. In general, this is close to impossible to reach in a meeting of the governing body of the Treaty, because the specific interest of one country can be just the opposite of the proposing country. Nevertheless, some modifications have been approved recently, and they are already included in the current official version of the Treaty, valid since 6 July 2020 [1].

This deficiency was informally overcome by inviting the International Institute of Refrigeration [3] to run a technical sub-commission, called CERTE, to deal with the proposed technical changes and to review the problems that could appear in the interpretation of some technical requirements. Nevertheless, this is not equivalent to the work of a group of experts working under the mandate of the agreement. Besides that, it is worth citing the existence and work of the Transfrigoroute association [4], which provides a unique experience on the actual application of these matters and should have some role in assessing the technical proposals.

In the WP.11 official meeting of 2020, the Vice-Chair of the Working Party presented a critical review of the prospects of ATP, highlighting that major developments may be expected in the text of the Agreement in the near future, mainly due to environmental reasons. He proposed paving the way for redefining ATP according to new regulations, which may require significant changes and a full reconsideration of the text and technical annexes of the Agreement [5].

That speech was a very good approach to the foreground of ATP. It points out the coexistence of different types of problems, starting with the definition of perishable foodstuff, which is not a unique class of products, and arriving at the possibility of continuous and complete surveillance of the temperature and other conditions of the transported merchandise, fully online. A high-quality market will pull from this sector in that direction. This paper is intended to make a contribution to the physics of the systems that are the roots of this activity, and about the technology that could answer the new requirements. A different perspective is related to the legal and binding clauses [6,7,8] but this is not considered in the paper. Other important aspects relate to geography and climate because it is obvious that the difference in latitude has a strong influence on the working conditions of the ATP units [9,10,11]. On the contrary, an important point that must be taken into account in this work is innovations [12,13], although this field does not advance significantly because of the stiffness of the Agreement.

**Figure 1:**
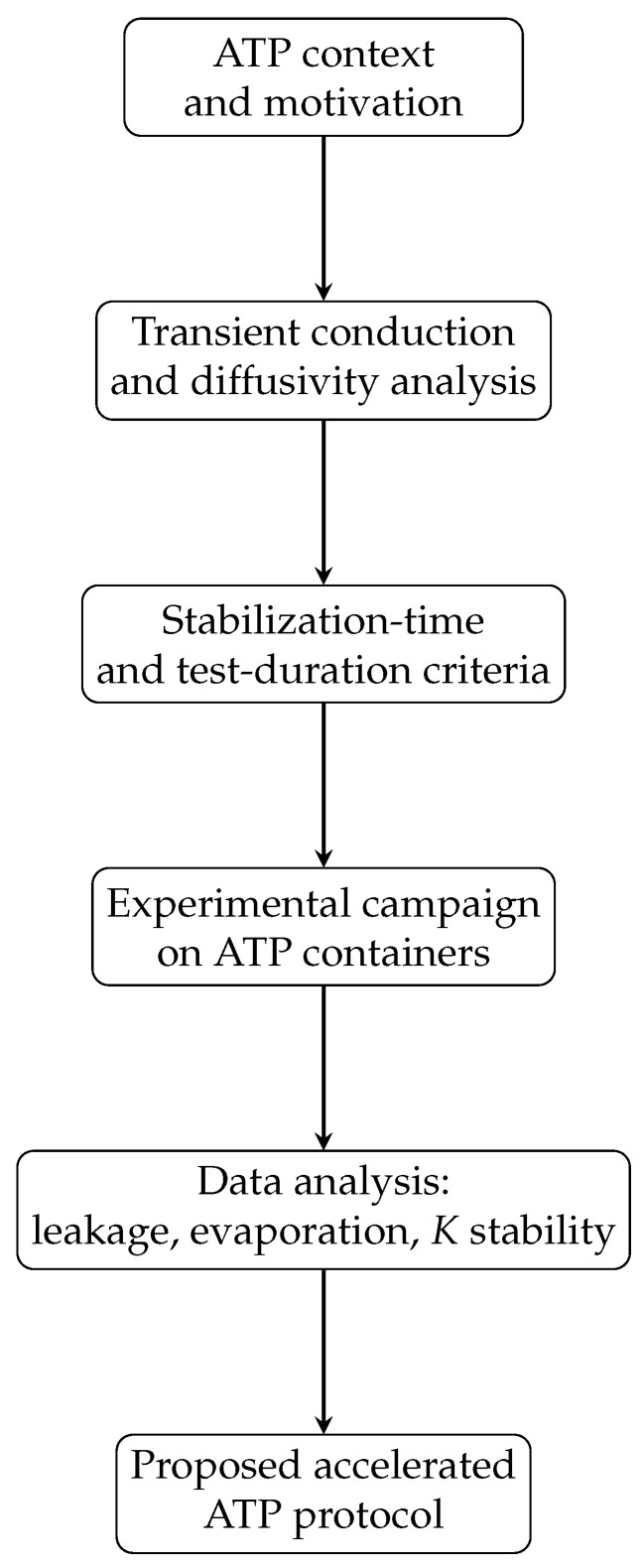
Methodological framework followed in this work, combining theoretical analysis of transient conduction with experimental tests and protocol design.

The approach followed in this paper (see Figure 1) is to start from scratch, because the basic test established by the ATP, the so-called isothermal test, is not actually rooted in a sound foundation. Requirements are prescribed without any justification, and many of them can be questioned. For instance, temperature recordings were prescribed every 15 min, which is too long for characterizing a thermal transient. On the contrary, a steady state must be kept for almost 24 h, which, again, is too long and expensive, and does not add any statistical significance to the measurements, because they are simply repetitive, and they only guarantee that the test station can maintain constant boundary conditions for 1 day, but nothing is learned about the performance of the box, tank or container being tested.

Although several studies have characterized the thermal behavior of insulated containers in the context of ATP testing [14], the standard procedure has remained essentially unchanged for decades. Existing literature does not address the statistical limitations of using a single steady-state measurement, nor the influence of transient conduction, chimney-driven air leakage or latent-heat effects on the measured *K* value. Consequently, there is no established methodology for reducing testing time while improving accuracy.

This work fills that gap by (i) developing a transient-based analytical framework to estimate stabilization times using dimensionless criteria; (ii) performing extensive experimental tests on commercial ATP containers to identify and quantify secondary physical phenomena; and (iii) proposing an experimentally validated accelerated protocol that shortens the test duration and reduces uncertainty, while remaining fully compatible with the ATP classification requirements (IN and IR).

Optimizing the ATP isothermal test is not only of academic interest but has clear industrial and regulatory implications. The number of units requiring periodic ATP verification is steadily increasing, and long test durations translate into higher costs, reduced availability of certified equipment and logistical bottlenecks at test stations. A scientifically grounded, time-efficient protocol that maintains or improves accuracy can therefore benefit test laboratories, fleet operators and regulatory bodies alike.

The next section develops the theoretical framework that underpins the proposed optimization, combining classical heat-transfer principles with original analytical approaches.

## 2. Theoretical Framework for Thermal Transients

### 2.1. Principles of Heat Transfer

Heat-transfer fundamentals—conduction, convection and radiation—have been well-established since Fourier’s seminal work in 1822 [15]. These principles underpin the analysis of thermal transients in insulated containers, which is central to ATP qualification. Rather than reviewing the general theory, this work applies it to the optimization of the isothermal ATP test, building on previous studies in this context [14].

The present approach focuses on transient conduction and thermal diffusivity as the parameters that govern stabilization time, combining these concepts with experimental evidence to propose a shorter and more informative protocol. For comprehensive treatments of heat-transfer theory, standard references such as Levenspiel [16], Stoecker [17] and Jaluria [18] may be consulted. The following sections therefore address only the analytical framework and experimental results developed by the authors.

### 2.2. Revisiting an Old and Simple Case: Analysis of Thermal Transients in a Slab

Although an infinite slab made of a uniform material is the simplest case found in heat transfer, and can easily be solved by numerical computing—and it was formerly solved graphically (by Heisler)—this case represents quite well the actual problem of the ATP basic classification, related to the “Isothermal” label.

A box, a tank or a container (a unit, in general) is qualified as IR (Reinforced Isothermal) if its global coefficient of heat transfer from outside inwards (or the reverse, depending on the temperatures inside and outside that unit) is not higher than 0.4 W/m^2^ °C. If it is higher than that value and smaller than 0.7 W/m^2^ °C, it is qualified as IN (Isothermal Normal).

Said coefficient is measured as the following ratio, where *Q* is the total heating power released inside the unit in order to maintain a difference of temperature of 25 °C, between the inside and the outside. The test is performed just in reverse of the current commercial practice, where the inside must be cooler than the outside. For the test, it is much simpler to heat the inside. So, the overall heat-transfer coefficient, *K*, is given by

(1) $$K=\frac{Q}{S\cdot \left(T_{i}-T_{e}\right)}$$

Measurements must be performed in steady state, and the ATP is required to keep it for an exaggerated and useless time span, that is just the demonstration of the laboratory capability to keep constant temperatures as boundary conditions, but nothing else. ATP also requires the following values of temperatures, with some margin around it: $T_{i}=32.5\;{}^{\circ}$C and $T_{e}=7.5\;{}^{\circ}$C, and *S* is the representative surface of the whole unit; it is the geometric average of the internal and external surfaces of the complete wall [19].

The main practical objective of this work is to reduce the time span required to declare that the tested system is in steady state, and the cited measurements can determine coefficient *K*. Someone could say that it is not a very scientific challenge, but the question is that an enormous number of hours are devoted to making those measurements, which conveys higher prices for the test. It is really an industrial challenge because some governments (including Spain’s) have changed the domestic technical regulations on this type of transportation, and now require that all units with an ATP certificate have to verify their qualification in an official test station, when the units become 15 years old, and they must repeat the verification 9 years later. In Spain, there are more than 10,000 units turning 15 in a year, and the current test stations hardly could do the job if each experiment lasts more than 24 h. So, there is an enormous interest in reducing the length of the test.

Besides that, our analysis will show some peculiarities of thermal transients that have some scientific relevance. They are related to Fourier expansion in harmonic functions and can be used for interpreting the thermal evolution under the light of self-similar solutions. We will point out some important differences in the transient evolution speed for space-dependent functions that are very similar, as a cosine and a second-order inverse parabola.

About steady states, it must be recalled that they require some heat sources to keep gradients of temperature in the system. Otherwise, the only admissible solution is temperature uniformity.

In summary, this section is intended to have an analytical view of the problem addressed in the basic ATP tests. Such a view is expected to produce a qualitative but relevant picture of the physical problem behind our need to characterize the thermal performance of an ATP unit.

The concept of steady state is particularly well treated by Fourier, whose 1st theorem in the cited book, chapter II, section VI, says the following: “if all points within a homogeneous solid filling the space defined by six rectangular planes have temperatures following the linear equation:

(2) V=A−a·x−b·y−c·z

and the molecules at the six surfaces remain as such, and their temperatures also follow the given equation, all molecules inside this solid will keep their temperatures if there is not any change in the state of the solid by any external cause”

This theorem is directly derived from the time-dependent conduction equation, which is expressed as follows, in one-dimensional planar geometry, as is the common case for studying the wall of a chamber:

(3) $$\frac{\partial T}{\partial t}=\alpha \cdot \frac{\partial^{2}T}{\partial x^{2}}$$

α is the thermal diffusivity, and the main relation of this equation is that the time-dependent variation of *T* depends on the concavity or convexity of the profile of *T*. If the shape is planar (straight line) the second derivative vanishes, and the time derivative also vanishes. This is Fourier’s theorem, which was not taken properly into account when ATP was written and its equations were adopted. ATP considers the situation to be stabilized if the difference between inner and outer air temperatures does not change in more than 18 h. There are some complementary requirements about the uniformity of the temperature inside and outside the unit. Still, the most inconvenient feature is the cited one, related to the time span for making a test.

As the unit was already in a steady state at the beginning of this long period of 18 h, and the verification of being in such a state lasts less than 1 h (as will be shown afterwards) the test does not add anything to the knowledge of the unit. The test is just a confirmation of the capability of the test hall to keep the boundary conditions (which is a very simple task if the test hall has an air refrigeration system powerful enough). This is why some proposals are needed to properly focus on the ATP isothermal test, and this is the root of this paper.

Going back to Fourier’s 1st theorem, it has to be considered under the principle of isothermalization of a system. As a principle of Physics, all elements in a system will evolve to have the same temperature. The only way to have a steady state with different temperatures at different points is by the action of heat sources and heat sinks, which act in many cases as boundary conditions.

The problem of the time needed for a wall to reach the same temperature inside as the temperature of the fluid around it becomes a fundamental piece of this task because it is a measure of the time needed to reach a steady state (and make a test).

Numerical computation is the current and common way to calculate the performance of engineering systems, particularly in thermal engineering. This is especially needed if high accuracy is required, but it is less so if the objective of the calculation is to characterize the physics of the phenomena interplaying in the problem under study. For this purpose, analytical methods can provide better tools to look inside the Physics that we want to characterize. In our case, we will look for “Self-similar solutions” in time-dependent heat transfer, to point out the features of the problem.

It is well-known that the time-dependent equation of thermal conduction in one dimensional planar geometry (3) relates the time evolution of the temperature in a given point to the Laplacian at that point. The structure of the equation allows us to define a non-dimensional variable, called Fourier number Fo, defined for a slab of thickness H as

(4) $$Fo=\frac{\alpha \cdot T}{H^{2}}$$

This property points out that the concept of time and the space variable can be managed together, which can lead to self-similarity in the study of transients and can help formulate adequate criteria for running the test.

In his groundbreaking book, Fourier used his invention of expanding any function in terms of harmonic trigonometric functions (sinus and cosines of increasing frequency) to explain the temperature distribution inside a wall or similar body and its evolution. The method was completed years later by introducing eigenvalue equations obtained by variable separation in the temperature (T(x,t)=X(x)·τ(t)), which is a correct and systematic way to complete an analysis of the time evolution of temperature in a piece of material. Fourier also used other mathematical techniques for studying the complex subject of heat transients, but nowadays, the main route to solve a given problem of thermal conduction is by numerical calculation. This methodology is very powerful for obtaining accurate results of a given problem, but it is not so useful for depicting a general description of the phenomenology involved in the transients.

A mathematical technique which is implicit in Fourier’s analysis is the self-similar solutions approach, which is based on a well-known property of sinus and cosines, namely that their second derivatives have the same shape as the original functions (with a minus, and multiplied by the constant squared affecting the argument of the sinus or cosine). For instance, a function F(x) can be developed in Fourier terms as given below (for the sake of simplicity, function F has been chosen in this analysis as a symmetric one, defined in the 1D planar geometry between −H/2 and H/2). This function stands for the initial condition of the temperature inside the wall:

(5) $$F\left(x\right)=\sum_{n=0,1,\ldots }T_{n}\cdot cos\left(\left(2\cdot n+1\right)\cdot \frac{\pi \cdot x}{H}\right)$$

$T_{n}$ are the amplitudes of each harmonic (or mode), which are determined by using the orthogonal property among modes, namely

(6) $$T_{n}=\frac{\int_{-H/2}^{H/2}F\left(x\right)\cdot cos\left(\left(2\cdot n+1\right)\cdot \frac{\pi \cdot x}{H}\right)\cdot dx}{\int_{-H/2}^{H/2}cos^{2}\left(\left(2\cdot n+1\right)\cdot \frac{\pi \cdot x}{H}\right)\cdot dx}$$

Amplitudes characterize the importance of that mode in the function *F*, and they depend on the shape of the function. A function with significant importance of many modes is the uniform distribution, F(x)=c. Their amplitudes follow the law

(7) $$T_{n}=\frac{4\cdot c}{(2\cdot n+1)\cdot \pi }\cdot {\left(-1\right)}^{n}$$

Table 1 shows the amplitudes of the fundamental mode (*n* = 0) and the next 5 (with c = 1). It is worth noting that the fundamental mode is the only one which is non-negative in the full domain. It can be seen that the reduction of the importance goes inversely to linear (i.e., smoothly)

Much higher modes still have some importance in this expansion. For instance, $T_{10}$ is 0.0606 and $T_{20}$ is 0.0311.

**Figure 2:**
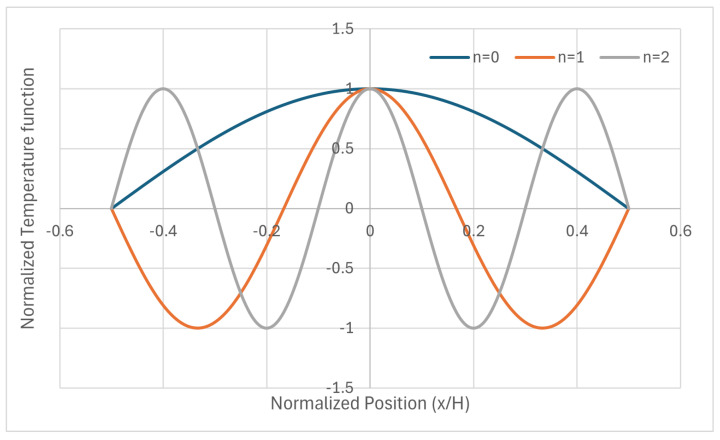
Modes *n* = 0, 1 and 2 for a one-dimensional wall (or slab) normalized to 1.

Figure 2 shows the fundamental mode of the “wall” case under study plus the following ones: *n* = 1 and *n* = 2. In the picture, they are represented with the same amplitude (=1).

A function that better describes the most common thermal situation of a wall is the parabolic shape, namely

(8) $$F\left(x\right)=1-{\left(\frac{x}{H/2}\right)}^{2}$$

This shape can correspond to a wall that is hotter and is cooling down by external convection. The maximum value of F is 1, and it goes to 0 at ±H/2. From the point of view of algebra, this is quite correct, but it is not so from the point of view of Physics, because of the boundary conditions and the thermometric scale being used. What does it mean that the temperature vanishes at both boundaries? Before approaching this topic for both functions selected for this study, it is worth recalling that the amplitudes of successive modes in the parabolic shape are given by the law

(9) $$T_{n}=4\cdot {\left(\frac{2}{\left(2\cdot n+1\right)\cdot \pi }\right)}^{3}\cdot {\left(-1\right)}^{n}$$

The summation of the amplitudes given in the Table 2 amounts to 0.9997, very close to 1, which is the true value of the function for x = 0. Indeed, parabolic shapes, which are very common in Laplacian problems, are very well represented by Fourier’s expansion. In fact, the addition of the fundamental mode plus *n* = 1 and 2 produces a representation of the parabola better than 1% everywhere, except in a small zone very close to the boundaries. In spite of this similarity, the parabolic profile and the cosine have very different consequences for the transient process. In a parabola, the partial time derivative of the temperature is the same for all points, because the second derivative of the parabola is constant. On the contrary, the second derivative of the cosine is a cosine itself (with a minus, because it conveys cooling when concavity faces downwards).

#### Boundary Conditions and Dominant Phenomena

The foregoing shapes of F(x) represent two extremes of the problem. In the case of the uniform function, F(x)=1, there is not any internal thermal flux, because the gradient of T is 0 in the full domain. In the borders, there is a jump in temperature, which is associated with a very low convection coefficient.

If we assume that the wall is surrounded on both sides by a fluid at a lower temperature, the isothermalization process will start as the uniform shape becomes bent, in order to establish the fundamental boundary condition, so

(10) $$-k\cdot {\left(\frac{\partial T}{\partial x}\right)}_{b}=h\cdot \left(T_{b}-Tf\right)$$

The parabolic shape is indeed very appropriate for being studied under Fourier’s method, but some explanation is still needed about the boundary conditions. First, we should take the temperature of the fluid $T_{f}$ as 0 in our thermometric scale. For the boundary condition to be applicable, the physical temperature in the actual physical boundary must be above 0, for the convection film to remove the heat. Obviously, the higher the convection coefficient, the smaller the temperature in the wall surface. At the same time, the slope of the temperature distribution inside the wall will have to follow the last equation, and this will be transmitted to the rest of the body, producing the thermal fluxes that will tend to isothermalize the wall with the surrounding fluid.

It is worth noting now that isothermalization implies heat transfer through the fundamental mode (n=0) from the hotter body (the wall) into the colder body (the fluid). However, for higher modes (n>0), isothermalization is mainly an internal redistribution of heat. Note that a mode has (2n+1) subdomains where the sign alternates between positive and negative values. So, for a high mode, most of the heat transfer is based on passing heat from the positive subdomains to the negative ones, which makes the process very fast.

**Figure 3:**
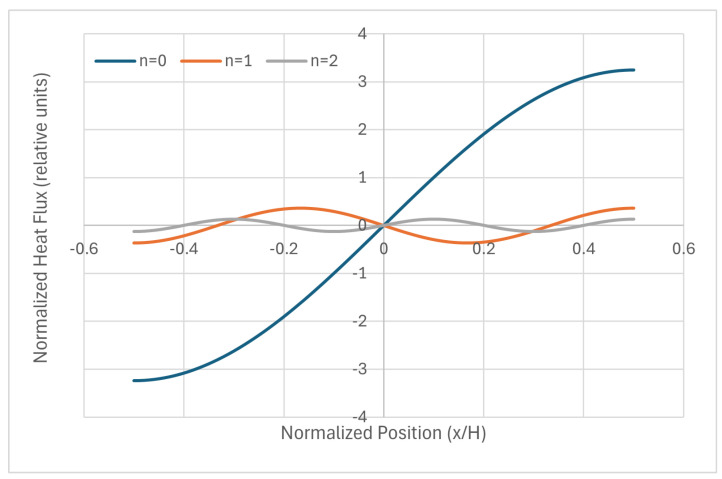
Thermal fluxes of each mode, in the parabolic case.

Thermal fluxes inside the wall are depicted in Figure 3 for the fundamental mode plus n=1 and n=2. They are related to the parabolic case formerly presented, and they obviously include the amplitudes of each mode, given in the former table.

### 2.3. Cooling Speed and Self-Similarity

The time-dependent conduction equation clearly states that the cooling speed just depends on the diffusivity and on the convexity of the geometric shape. A uniform distribution does not undergo any temperature decrease (but the system is unstable, because convection will induce a cooling in the borders, which will move inwards). On the contrary, a parabolic shape conveys a uniform cooling intensity, because its second derivative is the same everywhere. However, it does not lead to a self-similar evolution, because the evolution of the temperature is faster in the zones for smaller temperatures. If the system is considered homogeneous in physical properties, including the thermal diffusivity, the cooling intensity (J) can be defined as

(11) $$J\left(x\right)=\frac{\left(\frac{\partial T}{\partial x}\right)}{T}=\frac{\alpha \cdot \nabla^{2}\cdot T}{T}$$

For the parabolic distribution presented above, it is satisfied that

(12) $$J\left(x\right)=-\cdot \frac{8\cdot \alpha }{H^{2}-4\cdot x^{2}}$$

At x=0, the intensity is −8/H2. At the boundary, it tends to infinity, because the denominator is 0. In fact, it was already said that such a value could not be accepted, because of not being compatible with the boundary condition. Before going further in that direction, we should recover Fourier’s expansion methodology. If the cooling intensity concept is applied to any of the cosines that appear in this problem (the “wall” problem), it is found that

(13) $$J\left(x\right)=-\alpha \cdot {\left(\left(2\cdot n+1\right)\cdot \frac{\pi }{H}\right)}^{2}=J_{n}$$

As the cosine and its second derivative have the same shape, with a negative sign, which in this case means cooling, the intensity $\left(J_{n}\right)$ is the same at any point and at any time for each mode, which allows us to write a cooling general equation, in its way to isothermalization. The equation is applied to the central point of the wall abscissa, which is normal to the sides of the wall, but is the same for any point, for a given mode. We thus use the mode amplitude $T_{n}$ to characterize the cooling, and we can write

(14) $$\frac{dT_{n}}{dt}=J_{n}\cdot T_{n}=-\alpha \cdot {\left(\left(2\cdot n+1\right)\cdot \frac{\pi }{H}\right)}^{2}\cdot T_{n}$$

Therefore,

(15) $$\frac{dT_{n}}{T_{n}}=-\alpha \cdot {\left(\left(2\cdot n+1\right)\cdot \frac{\pi }{H}\right)}^{2}\cdot dt$$

(16) $$T_{n}\left(t\right)=T_{n}\left(0\right)\cdot \exp\left[-{\left(2\cdot n+1\right)}^{2}\cdot \alpha \cdot \frac{\pi^{2}}{H^{2}}\cdot t\right]$$

It is clearly seen that higher modes decay much faster than the fundamental one, which gives the isothermalization time. It is worth writing this equation with the Fourier number, $F_{o}$, defined in Equation (4):

(17) $$T_{0}\left(t\right)=T_{0}\left(0\right)\cdot \exp\left[-\pi^{2}\cdot F_{o}\right]$$

The cooling time can thus be expressed in terms of non-dimensional Fourier number, as the time needed to reduce $T_{0}$ by a given factor. For instance, the reduction of $T_{0}$ to 0.01 $T_{0}\left(0\right)$ requires $F_{o}=0.467$; and the reduction to 0.001 needs $F_{o}=0.7$. For instance, if the diffusivity is $10^{-6}$ m^2^/s and H=0.1 m, the time needed to reach $F_{o}=0.7$ is 7000 s (close to 2 h). If a reduction of 0.01 is enough to consider thermalization, the time required is 4670 s (1 h and 16 min).

It must be added that some authors choose H/2 as the characteristic length for defining $F_{o}$. If that recipe is followed, it holds

(18) $$F_{o}=\frac{4\cdot \alpha \cdot t}{H^{2}}$$

The result in time is the same, but not in Fo.

**Figure 4:**
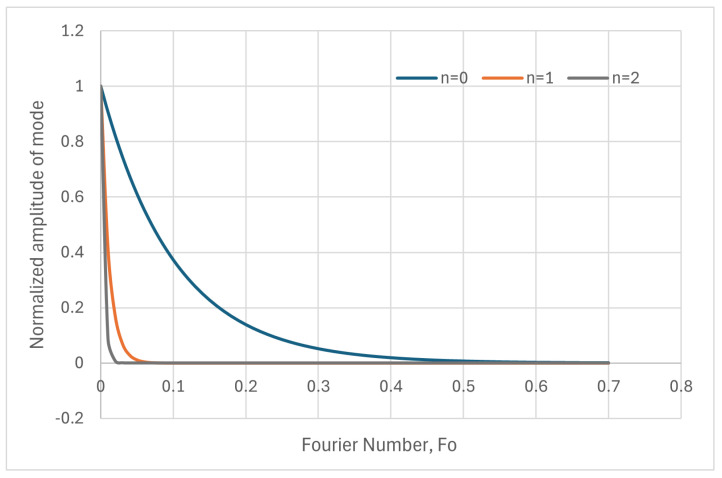
Time-dependent evolution of the amplitudes of the fundamental mode first and second harmonic (it is measured in relative terms; of course, the initial values are not equal to 1 in both modes; actual amplitudes at t = 0 are given in Table 1).

Figure 4 shows the evolution of the amplitude of the fundamental mode, versus Fourier number. Exponential decay is clearly observed, which is much faster for the second harmonic (n = 2). The transient can be considered finished at $F_{o}=0.02$ for n = 2, $F_{o}=0.05$ for n = 1 and at $F_{o}=0.5$ for n = 0.

**Figure 5:**
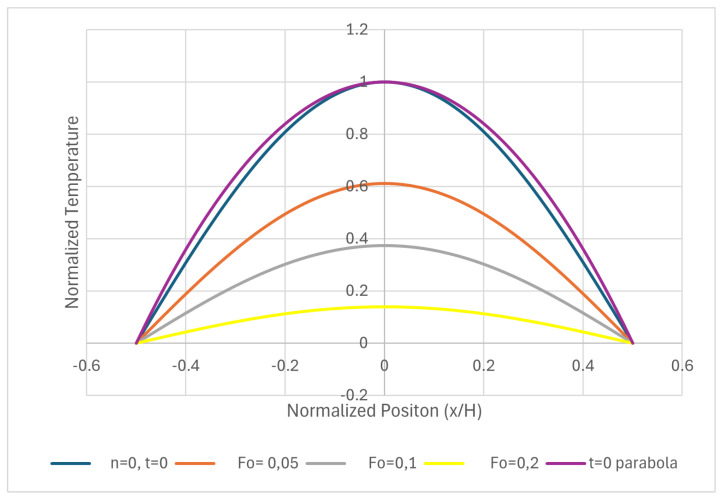
Shapes of the self-similar evolution of the temperature inside a wall with a parabolic profile as an initial condition. The fundamental mode is also depicted for t = 0, as well as said mode along time (measured in terms of Fourier number).

Self-similarity of the profile of temperature inside the wall can be appreciated in Figure 5.

#### Boundary Conditions: Physical Wall and Virtual Wall

Mathematical models do not always fit well with physical phenomena, particularly in relation to boundary conditions. In Mathematics, said conditions are usually expressed in terms of the main function being studied, setting some values for the function at each border. Of course, mathematical expressions for boundary conditions can embody the value of the first derivative just at the boundary, to involve the flux. This fact can complicate the mathematical apparatus, and it can even jeopardize the validity of some mathematical properties, such as orthogonality. In the case of cosines, the best boundary conditions are T=0 at the boundary, which creates a problem in the Physics of the problem, because this means that heat cannot be transferred from the wall to the surrounding fluid (which was adopted as a reference for 0 degrees). The main boundary condition in our case is

(19) $$-k\cdot {\left(\frac{\partial T}{\partial x}\right)}_{b}=h\cdot \left(T_{b}-0\right)$$

**Figure 6:**
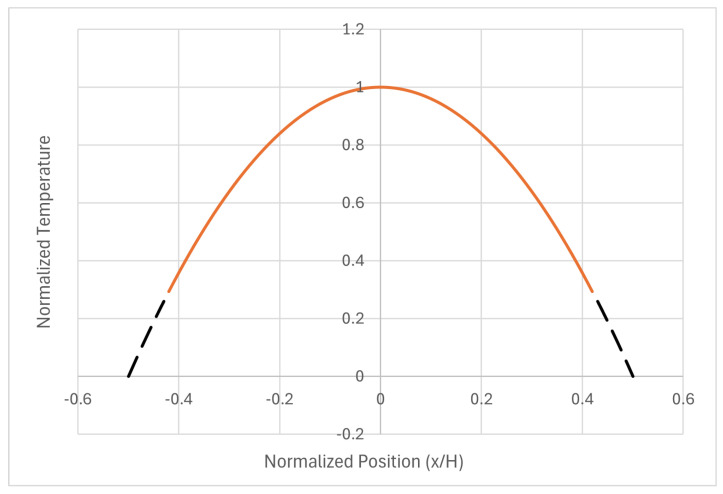
The physical wall covers from −H/2 to H/2, and the virtual wall, where mathematics is formulated, goes from −V/2 to V/2.

So, if $T_{b}$ is 0, there is not any way to use the model, because it is blocked. However, there is a simple trick that can be used to reach a compatible solution between Physics and Mathematics, and it is based on adopting the actual wall as the true system for Physics, and a virtual wall for Mathematics. Both systems are depicted in Figure 6.

Relations between both systems can be derived from an analysis of the boundary condition, as expressed above. In the actual border, the function has to have a temperature, and its derivative has to have a value that meets the equality of the former condition, which implies that the heat arriving at the physical border by conduction is extracted exactly by convection of the surrounding fluid. It holds

(20) $$k\cdot \frac{\pi \cdot T_{o}}{V}\cdot sin\left(\frac{\pi \cdot H}{2\cdot V}\right)=h\cdot T_{o}\cdot cos\left(\frac{\pi \cdot H}{2\cdot V}\right)$$

In order to write the equation using the Biot number, Bi, which is an ordinary reference in this field, we can recall

(21) $$Bi=\frac{h\cdot H}{k}$$

We call m=V/H, and so we find the relation between both systems, which is

(22) $$tan\left(\frac{\pi }{2\cdot m}\right)=Bi\cdot \frac{m}{\pi }$$

**Figure 7:**
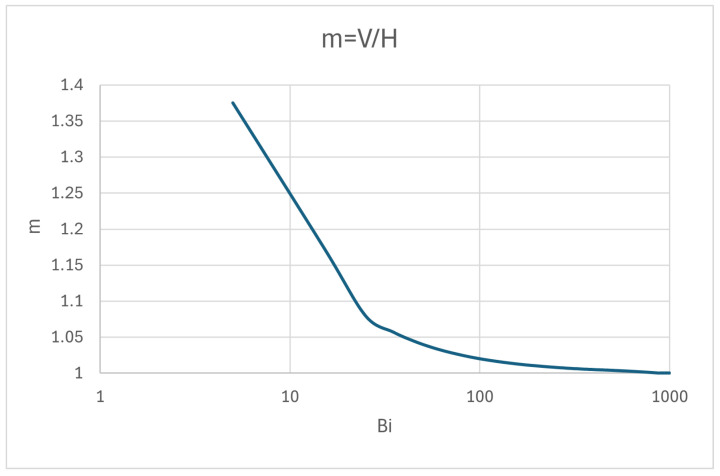
Ratio of thickness between virtual and actual wall, depending on Bi.

Figure 7 shows the dependence of m on Bi. It is obvious that very low h values lead to higher separation between both systems. Moreover, the methodology formerly described can be used to calculate very accurately the cooling features of a hot wall surrounded by a cooler fluid and gives a good estimate of the time needed for the cooling transient to take place.

### 2.4. Assessing the Cooling of a Wall

**Figure 8:**
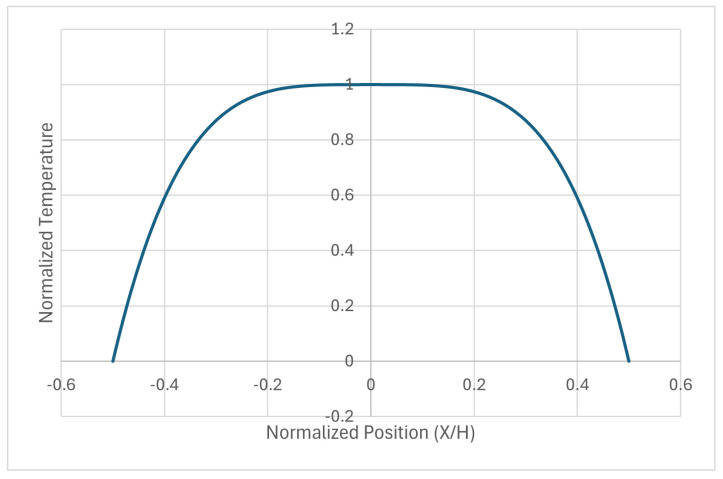
A temperature profile corresponding to $T=1-16\cdot x^{4}$.

A better representation of the initial conditions of a wall is given in Figure 8, where the temperature profile corresponds to a fourth-order parabola. It is normalized to 1 in the center of the wall.

**Figure 9:**
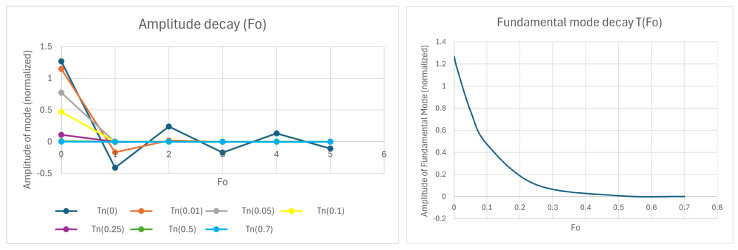
Cooling of a fourth-order parabola profile, measured as the corresponding amplitudes of different modes at different times (time is represented by Fourier number, $F_{o}$).

Figure 9 shows the time decay of the amplitudes of the main modes, as a function of $F_{o}$, which is the right way to measure it. In the figure on the left-hand side, it is seen that the amplitude of the higher modes vanishes very rapidly. The time evolution of the fundamental mode is shown in the figure on the right-hand side, and it can be seen that $F_{o}=0.5$ marks the end of the transient. In the following, a numerical criterion will be developed to determine when a steady state has been reached, which will always need a heat source acting in some part of the system. Otherwise, the system will become isothermalized with the dominant surroundings.

Figure 9 is relevant for defining the end of a transient in a wall, which is dominated by the decay of the fundamental mode. Although there is not a black/white threshold in this evolution, which follows a smooth analytical law, a Fourier number of 0.5 seems to be an acceptable value to consider that the transient has finished. Moreover, this figure is only a qualitative indication of the duration of the transient, but the test’s final measurements will have to be performed once the steady state is achieved, which could be defined as a long period of time with constant measurements, where constant values mean that their variations are not larger than the uncertainty or error bar allowed in the ATP technical annex.

### 2.5. Numerical Recipe for Transient Times

**Figure 10:**
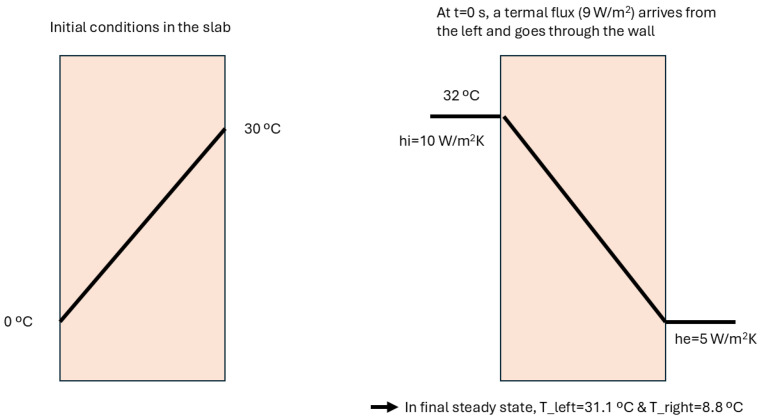
Initial and final conditions of a wall of an ATP box.

**Figure 11:**
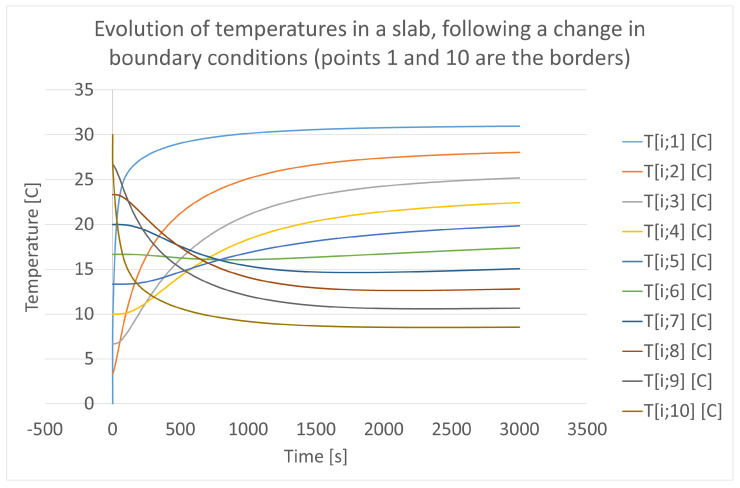
Evolution of temperatures at both faces of the slab of Figure 10 plus 8 internal points with equal separation: point 1, left face; point 10, right face.

A physical situation relevant to the problem of featuring the thermal performance of an ATP container is the one depicted in Figure 10 and Figure 11. In the former, a wall is represented with two different linear temperature profiles: the initial one, on the left-hand-side, and the final state, to the right. It represents a case of the wall of an ATP box that has been loaded with a cold foodstuff in a day with outer air at a warm temperature, and it suddenly changes in the test hall of an ATP station with warm air inside and cold air outside the box (inside the hall).

This figure is very helpful in illustrating the geometrical features of a transient with a double origin: heat incoming from the left and heat outgoing through the right. The final state of this theoretical experiment is very simple to calculate analytically and gives surface temperatures of 31.1 °C on the left, and 8.8 °C on the right. (Note that the final state is not symmetric because of the different values of the convection coefficient h.) It can be seen in this figure that the temperatures at both faces are 30.9 °C and 8.74 °C, for a time of 3000 s. This means that the changes at this time are over 99 % of the total change at the very end of the transient. Moreover, the experiment is accepted with an error of 1 %, which in this case corresponds to 0.25 °C. This value is higher than the variations of temperature still to be undergone in both faces, which are 0.2 °C and 0.06 °C. Indeed, the transient can be considered finished at that time, which is less than 1 h. However, it is worth noting that internal points are still evolving with a slightly higher speed (of temperature variation) and this magnitude reaches 0.0009 °C/s, equivalent to 0.05 °C per minute in the central points. Those points are the last ones in achieving stability because they are far from the faces where the new boundary conditions have been implemented. Moreover, those values are also very low and do not directly affect the experiments. They are shown here in order to clarify the geometric features of a planar transient and to complement the explanation of this case. Note that this case can be used for estimating transient times in any slab of uniform properties. In the preceding calculation, the following material properties have been used:

- Conductivity = 0.04 W/m°C.
- Density = 50 kg/m^3^.
- Specific heat = 1.4 kJ/kg°C.
- Diffusivity = 57 mm^2^/s.

For slabs immersed in fluids with the same boundary conditions, the conduction part can be calculated with the result shown before, using the Fourier number for that purpose. Note that it can be written as

(23) $$F_{o}=\frac{\alpha_{0}}{H_{0}^{2}}\cdot t_{0}=f_{0}\cdot t_{0}$$

where subindex 0 means that those values are the reference ones of the foregoing problem. Two parameters can change from one case to another: diffusivity and thickness. In general, we can write

(24) $$\alpha =m\cdot \alpha_{0}$$

(25) $$H=n\cdot H_{0}$$

The $F_{o}$ number must be kept constant for comparing the same situation, and this leads to

(26) $$F_{o}=f_{0}\cdot \frac{m}{n^{2}}\cdot t$$

We can obtain the time to reach a given conduction situation, which is

(27) $$t=\frac{n^{2}}{m}\cdot t_{0}$$

This recipe features the variations of the conduction transient times, and it is seen that smaller diffusivities (m < 1) and thicker walls (n > 1) lead to longer transient times, but these ones are bounded to values of a few hours for ordinary materials and wall sizes.

Although the actual heat-transfer processes in an ATP container are inherently three-dimensional and spatially non-uniform, simplified one-dimensional or lumped representations are commonly employed to identify dominant time scales and to guide experimental interpretation, provided their limitations are clearly acknowledged [20,21]. In the present work, these simplified models are not used to compute the global heat-transfer coefficient directly, but to support the definition of stabilization criteria and the design of the experimental protocol. The implications and limitations of this approximation are further discussed in the Conclusions.

## 3. Experimental Analysis and Proposed ATP Protocol

This section analyzes natural-convection-driven air leakage mechanisms not as an isolated heat-transfer problem, but to explain why long-duration ATP tests are particularly sensitive to non-ideal physical effects and why reducing the testing time is a key objective of this work.

### 3.1. Experimental Results and Interpretation

**Figure 12:**
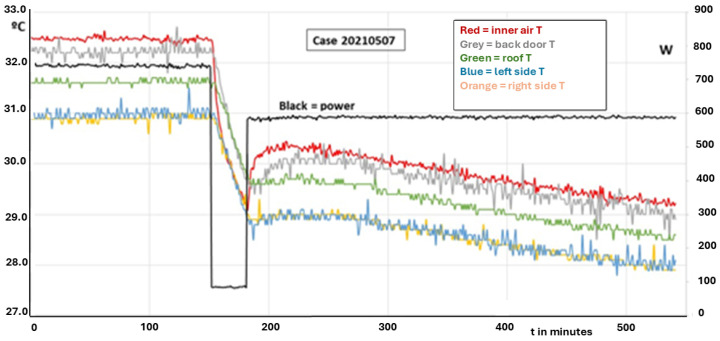
At the end of the steady state of an official ATP test, a transient is induced by modifying the value of the internal heating power. These transients are very important to properly characterize the thermal features of the container.

Scientific knowledge and technical applications must be based on well explained experiments that can be performed and repeated with a coherent theory that is also useful and applicable to other cases and scenarios. In this section, some experimental measurements will be analyzed for the sake of fully understanding the matter, which can be simplified once the relevant experiments are explained. Of course, our subject will be the isothermal test, which is the base of the ATP qualification. Figure 12 represents the end of the result of an official ATP test, followed by some phases aimed at characterizing the container under study. It is seen that at a given moment, t = 150 min in the time scale shown in the figure, the heating power generated (by electric ohmic heating) is set to 0 (from the previous value of 656 W), and only the fan power remains (which also contributes to heating, but it is only 84 W).

In the figure, the evolution of five different temperatures is depicted, corresponding to inner air; and four inner faces, corresponding to the back door, the ceiling and both lateral walls, right and left. These ones have the same evolution, all the time, which is not uncommon because they are made the same. In the first part of the picture, before interrupting the heating, both the total heating power and the temperatures were constant. It must be clarified that the ATP test was much longer in time, but the previous part does not have any interest for our purpose in this paper. In this case, it must be underlined that the temperatures of the lateral walls are lower than the rest of the temperatures, which is an indication that those walls are more powerful coolers than other parts of that ATP box. Take into account that the film coefficient inside the container will be fairly uniform, which means that the thermal flux incoming into a given part of the container wall will be proportional to the difference in temperature between the inner air and the surface of that given part. This indication is confirmed by the evolution of temperatures after cutting power, where those walls act as true heat sinks. Inner air becomes almost isothermalized with them, and other parts (ceiling, back door, …) follow the temperature evolution of the lateral walls. It must be noted that the temperature decay along the period without heating power is a little bit fast for a fluid (air) contained in a container that, theoretically speaking, only loses heat by transmission through the walls by conduction. This means that the only heat sink acting for cooling the inner air is the transient mechanism that we have analyzed in the foregoing paragraphs, and this mechanism is fully dominated by thermal diffusivity. In good thermal conductors, such as Al or Cu, this parameter reaches 100 mm^2^/s. Ceramic materials and common bricks have values around 1 mm^2^/s. Usual insulators (with low conductivity and low density) present values around 0.1 mm^2^/s. In Figure 12, we can read for the surface temperature of the lateral walls that T = 31 °C at t = 154 min, T = 30 °C at t = 167 min and T = 29 °C at t = 182 min. In the following sections, several experimental tests will be shown and the concept of well-behaved evolution will be introduced, as a result of the fundamental thermal principle that states that heat goes from hotter parts to colder ones.

A theoretical analysis can help understand the importance of relying on experiments to determine the K value characterizing an ATP item. Our method will be based on the decline of the air temperature when the internal heater is disconnected, once the system has arrived at a steady state. Following the capacitance method, it can be written as

(28) $$V\rho C\frac{dT}{dt}=\;-SK\left(T-T_{0}\right)$$

where V is the volume, ρ is the density, *C* is the specific heat, *S* is the surface of the item, *K* is the lumped parameter to classify the item in the ATP scale and $T_{0}$ is the external temperature, i.e., the temperature inside the laboratory hall where the test is performed. If we define the temperature difference

(29) $$\theta =\;T-T_{0}$$

the previous thermal balance equation can be rearranged as

(30) $$\frac{d\theta }{\theta }=-gdt$$

with

(31) $$g=\frac{SK}{V\rho C}$$

(32) $$\frac{\theta }{\theta_{0}}=e^{-gt}$$

If this method is applied to the red curve of Figure 12, after minute 150 and observing the T decay for 35 min (2100 s), the K obtained is 0.033 W/m^2^°C, which is an impossible value for any ATP item. In fact, real K was 0.4 W/m^2^°C. The error comes from a failure in the hypotheses of the case: a capacitance method can be applied to items without importance internal resistance. However, any ATP tank or box has a very large resistance because the thermal insulators are placed in their walls. So, the theoretical determination of K based on this model cannot be accepted.

### 3.2. Heat (and Mass) Transfer Through a Wall

An implicit hypothesis of the ATP Agreement is that ATP containers are hermetic. Therefore, the only way to transfer heat from inside to outside is by thermal conduction. However, ATP containers, including most of the tanks and vans, are not hermetic. Moreover, they could not withstand the pressure difference that appears when the mechanical equilibrium, which corresponds to equal pressure inside and outside, is distorted by heating or cooling one of those volumes (usually the inner part). Imagine a pressure bottle filled with air at the same temperature and pressure as the atmospheric air around it (let us say 27 °C and 100 kPa). Now, we heat the inside of the (closed) bottle by internal ohmic heating up to 57 °C. It is perfectly known (but it was somehow forgotten in the ATP technical part) that pressure will increase until 110 kPa, which means that a difference of 10 kPa will appear between the inside and outside. Such an overpressure is easily supported by a pressure bottle, but it would produce the explosion of some parts of the walls in a common ATP container. Only a few vans and tanks, mechanically reinforced, operate under hermetic closure. It should be noted that, before opening the doors, it is mandatory to equilibrate pressure by opening a relief valve. In ordinary containers, as soon as an overpressure appears, the walls are slightly bent, and very small channels appear in discontinuities of the pieces structured to make the container. Those channels mainly appear in the joints between normal plates, and they become larger as overpressure increases. In multiple cases, those small breaches stand forever once they reach plastic deformation. Besides micrometric channels mechanically produced, some insulators of ATP containers have some inherent porosity, because they are made of foams and expandable polymers. Porosity is a main issue in many oil and gas wells, particularly the latter, and they have been studied under different viewpoints and with different purposes, mainly in relation to moving fluid through a field of porous rocks. Similarly, hot air would go across a porous wall, producing an apparent thermal diffusivity higher than that of conduction [22]. To the authors’ knowledge, nobody has identified such a “combined diffusivity” as the actual one acting on an ordinary container. These phenomena modify Fourier’s heat-transfer general equation, which can be rewritten as

(33) $$\rho \cdot C\cdot \frac{\partial T}{\partial t}=k\cdot \frac{\partial^{2}T}{\partial x^{2}}+B\left(\frac{\partial P}{\partial x},\frac{\partial T}{\partial x}\right)$$

where B is a complex term involving pressure and temperature gradients. Tortuosity, which is a feature of the microchannels in porous media, conveys an enormous complexity that can only be characterized at a macro scale, but not at the detail of a differential equation that presumes a continuous material. This is a logical hypothesis when metallic materials are the subject because the metallic bond implies that the valence electrons move a lot around its main attracting atom. On the contrary, brittle materials (such as ceramics) and discontinuous compounds, such as glass fibers and wools, include porosity in their fabrication procedure, which in turn can be deployed in many different anisotropic distributions. The bibliography on porous materials is really very large, with a clear focus on geological scenarios, such as those of the hydrocarbon extraction industry [23,24,25,26,27]. The ATP scenario is very different from the geological ones. A classification that we propose in this paper is to distinguish between two types of micropores:

- “Valve” type pores, that remain closed if there is not any pressure difference between inside and outside. These are non-permanent micro-breaches that appear by mechanical deformation and do not show up if there is not any deformation, or it is very small. Once the mechanical equilibrium has been reached in a container with this type of pores, they do not contribute additionally to heat transfer, and the global heat-transfer coefficient does not vary if it is measured with different temperature differences.
- “Permanent” micropores or micro-breaches, that could be produced in the fabrication stages of the walls, or could be the result of hits and crashes, and poor maintenance. In turn, depending on the relative position and geometry, those micropores can remain inactive for a really long time; or they can become associated in some way, producing what we can call the “chimney effect”. This effect can become dominant and does not really stop when the mechanical equilibrium is reached, because it is part of that equilibrium; and the “chimney” must eject some mass flow to compensate the difference in air density at different temperatures.

**Figure 13:**
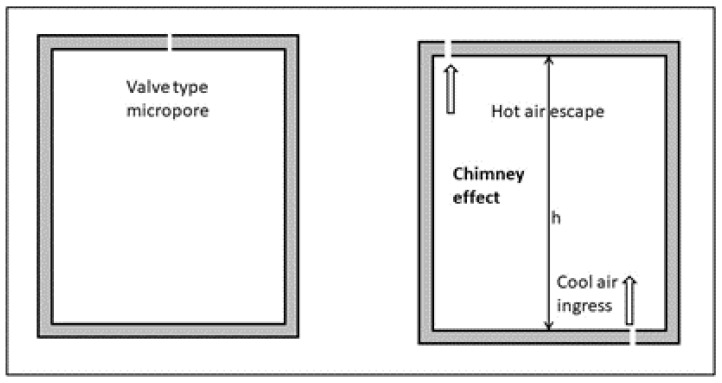
Two types of micropores can be distinguished in the walls of any container without strict hermeticity: valve type and chimneys. The former type has a main role in keeping mechanical equilibrium, equalizing both pressures, inner and outer. The latter has a bad effect on the thermal insulation features of the container.

A sketch of both types of pores is presented in Figure 13. The chimney effect is a particular case of Archimedes principle, and it generates a driving force, which can be expressed as a driving pressure that can be formulated as follows:

(34) $$\Delta P_{p}=g\cdot h\cdot \left(\rho_{c}-\rho_{h}\right)$$

This is a simplified view of the real problem because the active height producing the natural convection can have very different values depending on the geometrical distribution of the breaches, cracks and pores. Moreover, the former expression contains the elements of the pumping pressure, including g = 9.8 m/s^2^. This pumping will be equilibrated by the pressure loss along the full trajectory of the ejected air, which conveys a loss of thermal energy. The pressure loss is mainly produced when crossing the walls, and each contribution can be expressed as the product of three factors:

- The fluid regime factor, which is 64/Re for very low Reynolds numbers, in pure laminar regime, and is represented in general as a function of Re-m, m being 0.25 for the turbulent regime, which is not the case under study.
- The shape factor is usually represented as L/D, where L is the total length of the air track within the micropores and D is their hydraulic diameter. This factor is impossible to measure finely in most of the porous materials, but it can be adjusted to the results of several experimental observations.
- The dynamic pressure, $1/2\cdot \rho \cdot v^{2}$.

When the pressure loss matches the pumping pressure, the leakage speed v is theoretically determined, and the total escaping mass flow, m’, as well. An elementary approach to the problem is expressed by the following equation:

(35) $$\Delta P_{p}=g\cdot h\cdot \frac{P}{R}\cdot \left(\frac{1}{T_{e}}-\frac{1}{T_{i}}\right)=\Delta P_{loss}=f_{Re}\cdot \frac{L}{D}\cdot \frac{1}{2}\cdot \rho \cdot v^{2}$$

It is seen that the natural convection term (or chimney effect) produces higher leakage speed as the temperature difference (Ti – Te) increases. So, a characteristic of an ATP container suffering from the chimney effect is that the experimental determination of its overall heat-transfer coefficient leads to values increasing as the temperature difference does.

It is worth noting that the energy balance inside the container is now

(36) $$Q=h_{i}\cdot S_{i}\cdot \left(T_{i}-T_{w}\right)+m^{\prime }\cdot C\cdot \left(T_{i}-T_{e}\right)$$

The last term has been added to the classical equation used in ATP formalisms. The first term on the right-hand side is the heat-transfer rate across the walls. $h_{i}$ stands for the convection coefficient inside the container, $S_{i}$ is the internal surface, $T_{i}$ the air temperature and $T_{w}$ the temperature of the inner face of the wall. In the last term, we find the air temperature outside the container, $T_{e}$, the specific heat of air, C=1 kJ/kg·°C, and the mass flow escaping through the chimney.

**Figure 14:**
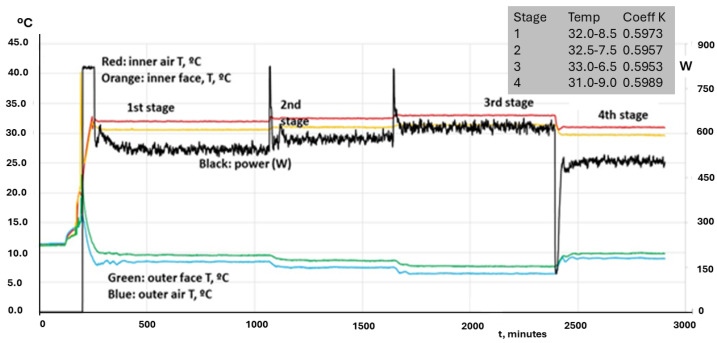
Four consecutive tests on the same box are carried out in this case. From one stage to the following one, heating power is changed, and so temperatures change as well. In this case, the box shows no chimney effect at all, and the K coefficient remains constant. In the graph, orange and green lines have been included, corresponding to the inner face and outer face of the walls: they are not used in ATP tests, but they are very useful to understand the underlying mechanisms. (Test case 20210526).

A test on a container, box, tank or van, which does not suffer from the “chimney effect” coefficient *K*, gives the same result if the experiment is carried out with different values of inner and outer air temperatures. Figure 14 shows the results of four tests on the same carriage, varying the range of temperatures. Each test is a stage of the same study, where Q is changed from one stage to the next.

Figure 14 is also useful to ask for a reduction in the test duration. ATP requires more than 18 h of constant values, which is something not rooted in any principle. In the figure, it is seen that our 4-fold test lasted almost 2 days (1 day = 1440 min) but each stage could be performed in much less time. After the starting phase to set the test hall in proper conditions, the steady state for determining the K value can be around 100 min, no more, even less. Note in this test that the rippling in power was very intense, while the temperatures remained quite constant. The reason for that is found in the way the experiment is defined. There is continuous feedback on the heating power to fix the inner air temperature at a given value (32.5 °C in the first stage). This method integrates practically all the physical information related to the inherent variations of the experiment into a single variable, namely the thermal power level (which has two components: the fan power to guarantee an air movement inside the box according to ATP specifications; and the heating power level, which is the variable modified to keep the inner air temperature at a given level).

The experiment reported in Figure 14 can be useful to illustrate this property. We will first restrict our analysis to the first stage, particularly in the range between 500 and 1000 (in minutes, recorded since the start of preparing the experiment). The fan power is 61 W and it remains constant. The average value of the heating power reaches 545 W and its standard deviation is 9.6 W. If normalized to the mean value, the latter becomes 0.0175.

We consider now the succession of temperatures (of inner air) in the same range, and we obtain an average value of 32 °C and a standard deviation of 0.031 °C, which becomes 0.001 after normalization. It can be seen that the temperature fluctuates very little (in fact, one order of magnitude lower than the heating power). It is therefore very important to analyze the rippling of the power both inside the first stage already mentioned, and the additional ones defined in the figure. Table 3 shows the average values, the standard deviation and its normalized value for the heating power and the temperature, for different ranges within the already defined first stage, in order to see if the increasing number of time steps selected in each case has a relevant influence on the accuracy of the method, or it is enough to record a moderate number of steps.

It is clearly seen that reducing the time span of the experiment, once the steady state has been reached, which happens before minute 500 (of total counting, not of actual test), does not convey a loss in accuracy for the temperature, even for the range 500–525. Of course, recording has been carried out every minute. At first, the information would be very sparse in time if the recording is carried out in much longer time steps. However, the physical system is very stable once the steady state has been reached, as well as the laboratory instruments. This conveys the possibility of enlarging the time steps without losing too much accuracy. For instance, if we take only the values recorded every five minutes, at 500, 505, 510, 515, 520, 525, we obtain the following values for the variables shown in Table 3: 537 W, 9.32 W and 0.017; and 32.017 °C, 0.041 °C and 0.0012.

Another important set of information is presented in Table 4, also related to Figure 14. In this case, the subject under study is the variation of the quality of the experiment when it changes from one stage to another. The information is structured as in the preceding table:

It is clearly seen that the full physical system, which includes all the elements from the unit under test, plus the instrumentation, plus the test hall installations, is very stable and robust, and can deliver the necessary information about the essential properties of the unit in a time span much shorter than the current one established by the ATP. The problem of defining an appropriate, accurate and robust experiment will be addressed in the next section.

**Figure 15:**
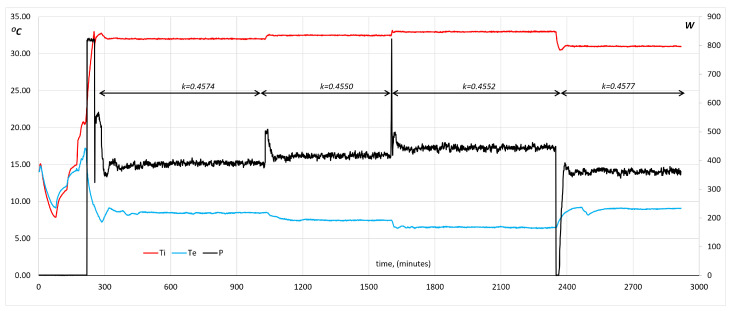
Another example of a case with four consecutive tests on the same box. From one stage to the following one, heating power is changed, and so temperatures change as well. In this case, the box shows no chimney effect at all, and the K coefficient remains constant. (Test case 20210531).

Another case with regular behavior is depicted in Figure 15. It shows that it does not suffer from the “chimney effect”, and coefficient K keeps its value across the swept domain, which is larger than the restricted domain expressed by the ATP technical annexes. This is an indication that the official ATP test can be shorter in time, and broader in temperature coverage.

The last case (Figure 15) is very useful for identifying the duration of the global transient produced by the change in the heating power level, which is not only seen inside the unit but also on the external side. It is worth pointing out that the test hall refrigeration system has to remove a different power level of heat in each case, which means that the blue and green lines in the lower band of the graph change to meet the refrigeration balance. This is also a measurement of the duration of the transient. For instance, moving from level 1 (400 W) to level 2 (425 W) at minute 1050 conveys a reduction of the test hall air temperature of 1 °C and it lasts around 150 min, but we cannot take this value as an absolute number, because it depends on features of the refrigeration equipment that can slightly change from a situation to another. For instance, the transient generated by the change from level 2 to level 3 (450 W) is faster and lasts around 40 min. The last change from 450 W to level 4 (375 W) is distorted by the operator, who is not able to fix the power level until a couple of hours later (for unknown reasons). Anyhow, the reaction of the full system is totally in accordance with the theory explained in the former paragraphs, and the final transient is again about 30 min long once the power level becomes fixed.

### 3.3. The Problem of Air Leakage

**Figure 16:**
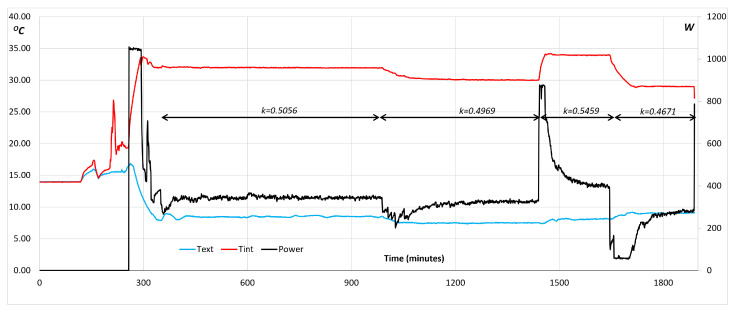
A 4-test case showing “chimney effect”. As presented in Figure 17, coefficient K reaches higher values as the difference between inner and outer temperatures increases. (Test case 20210505).

We come back now to wall porosity and chimney effect. Figure 16 shows a four-stage experiment of a box suffering from this effect. This is something that was not known beforehand but comes out of the experiment naturally. The unexpected mechanism is identified by its main effect, which produces larger heat losses as the temperature difference increases. This was evident from the balance equation, which is repeated here with some modification, in order to include coefficient K.

(37) $$Q=k\cdot S_{avg}\cdot \left(T_{i1}-T_{e1}\right)/\Delta H+m^{\prime }\cdot C\cdot \left(T_{i}-T_{e}\right)$$

where ΔH stands for the thickness of the insulating wall, and $S_{avg}$ is the effective surface of the wall, as heat conduction is concerned. It is the geometric mean between the inner and outer surfaces of the box, van or tank. We have used the property that all the heat transferred by conduction has an inner boundary condition that can be written as

(38) $$f_{1}=h_{i}\cdot \left(T_{i}-T_{w}\right)=\frac{k\cdot (T_{i1}-T_{e1})}{\Delta H}$$

Coefficient K is given by

(39) $$K=\frac{Q}{S\cdot (T_{i}-T_{e})}=\frac{k\cdot S_{avg}\cdot \left(T_{i1}-T_{e1}\right)/\Delta H+m^{\prime }\cdot C\cdot \left(T_{i}-T_{e}\right)}{S\cdot (T_{i}-T_{e})}$$

which can be approximated by

(40) $$K=\frac{k}{\Delta H}+\frac{m^{\prime }\cdot C}{S}$$

It must be remembered that $m^{\prime }$ is an increasing function of $\left(T_{i}-T_{e}\right)$ when a chimney effect affects the container. Therefore, the global coefficient K will also be a growing value with increasing temperature differences.

**Figure 17:**
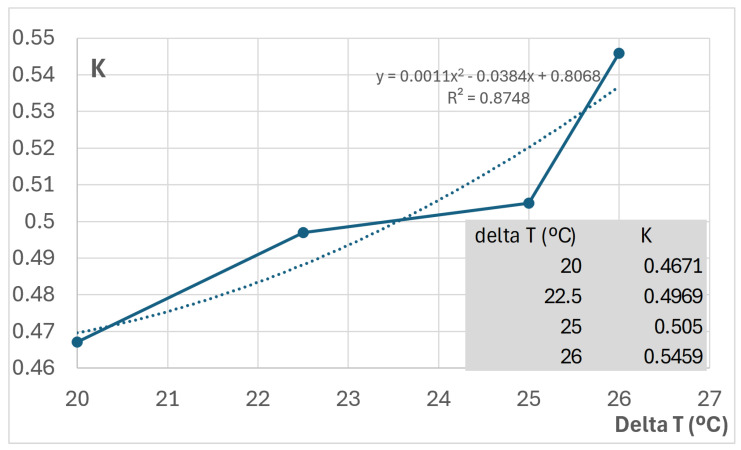
K values of the container tested in the experimental determination given in the previous figure. The dependence of K on the temperature difference between the inside and outside of the container is relevant. (Test case 20210505).

Figure 17 presents the dependence of the global heat-transfer coefficient on the temperature difference for this case that suffers from the chimney effect (natural convection). The line of tendency is also shown, and it corresponds to a parabola with a positive coefficient in the second-order term. It is worth pointing out that the regression coefficient is quite high (close to 1) with a value of $R^{2}=0.875$.

It could be said that these types of systems are not considered in the technical annexes of the ATP Agreement, but they are actual carriages, and they should be considered in some way in the Treaty. Of course, if any of these types of containers undergo the test, the coefficient K obtained will be the one corresponding to a temperature difference of 25 °C, but nothing will be added about the fact that K will be higher in working conditions with higher differences of temperature. Complete information on K would require more than one test (but they could be much shorter than the current ones, which are unnecessarily long).

**Figure 18:**
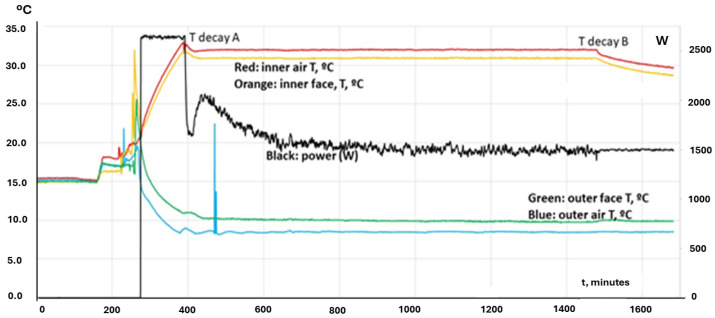
An example of a test with an additional operation of opening a very small slot in the rubber covering the frame of the door. Two different types of temperature decay are observed. Type A appears at minute 400 (the count of the experiment starts when the test hall opens, which is much earlier than the start of the experiment). There is a decay induced by the variation of the applied heating power in order to stabilize the air T around 32.5 °C. This is a direct reaction to the heating source. There is another T decay (type B) at the end of the test, which is not produced by changes in the heating power, which remains constant. This is produced by opening the door so that a small chimney effect is produced. (In general, doors are fully open at the end of the test, and there is a rapid removal of air and everything becomes isothermalized with the air of the test hall).

Container defects that can generate the “chimney effect” can be produced by standard use, because of deterioration of the rubber stripes closing their doors. Figure 18 is very relevant to that end.

After addressing the impact of air leakage and chimney effects, the next subsection proposes a revised ATP test protocol, supported by statistical criteria to ensure reliability and efficiency.

### 3.4. Proposed Protocol and Statistical Significance

It is evident that the current ATP test to determine coefficient K in isothermal tests is not in agreement with the scientific methodology for making experiments nowadays [28,29]. First, a better-explained connection between the physical model and the experiment is needed, and this includes prior work to analyze in depth the physical domain where the experiment has to be performed. Note that some hypotheses, such as the complete hermeticity of the boxes and tanks in ATP, must be fully discussed (and disregarded, in this case) as a previous step before the definition of the test.

Second, the test must have statistical significance compatible with the money and effort one can spend on the test. Of course, this depends on the decisions of the rulers of the market and the social and economic activity, but scientists and engineers must optimize their work within the design window so established. Statistics needs more than one measurement, which is the current situation. If only one measurement is obtained on a physical magnitude, there is no statistical significance at all. The degree of freedom in any statistical survey is the number of individuals minus 1. If our sample only has one measurement (one individual in this case), the degrees of freedom are zero.

Of course, one can use partial information about the instruments employed in the experiment for determining an error bar or a level of uncertainty, but this is not to be taken as a statistical significance of the system being tested. Even the application of quality standards, such as the EN17025, does not confer any statistical significance to the tested unit. This standard poses some requirements on the instruments used, to make sure that the test is measured with appropriate tools, but it does not require anything else to the definition of the test specified in the ATP. This specification is what must be revisited to define an updated and sound test.

In the current version of the ATP (and since its very beginning), the isothermal test has been performed according to the following specifications:

The tested unit is placed in a test hall, which remains closed during the whole test. Air is recirculated to remove all the heat load of the hall, thanks to intermediate heat exchangers that are connected to a refrigeration equipment placed out of the hall. Air in the hall must be around 7.5 °C (with some margin of change, but the air temperature must be kept constant).

Inside the unit, which is closed, an ohmic heater and a fan deliver heating power and a recirculating speed. Electric feeding is carried out through sinkholes and the like, which must be perfectly sealed once the cables are placed, including those of the temperature recorders. The air temperature inside the unit must be around 32.5 °C. It must be kept constant, with some margin of variation.

The test protocol is very lengthy, and can be summarized into two phases:

- Achieving the specified conditions, which can require a few hours, depending on the thermal inertia and the conditions outdoors.
- Keeping the steady state for 18 h, recording variables every 5 min. Before July 2020, it was 4 times an hour. It seems that the persons who took the decisions at the start of the Agreement consider that the evolution of the thermal transients was very slow.

In the preparation of this section of the work, we have mainly followed two books [28,29]. Both books have a sound background on statistics to determine error bars or uncertainties or to assess the significance of the measurements against a theory, which should be well-established.

In our case, heat transfer is a well-established science with a dozen of very good books teaching the same subject (with different flavors, but the same meal). Names such as Chapman, Mills, Incropera, Stoecker, Kaviany, Bejan, Jaluria and others constitute a fundamental library of thermal engineering, although we could go back two Centuries in History, to find Fourier’s “Theorie analyque de la chaleur” as the starting point of our subject.

Nevertheless, we should go down to the very specific problem under consideration, which is the ATP Agreement and its isothermal test. For reasons of continuity, we propose to keep the classification IN and IR, and the K values specified for each class, but the test protocol should be rewritten for better use of time and effort, and a better service to the public, particularly users of services delivered under the umbrella of ATP.

We must then come back to the equation of K, for paying attention to the thermal part. In general, variables under consideration in any experiment can be classified into two categories:

- Independent variables, or inputs into the system, where we can classify the electric heating power, Q. A fixed value can be used for a given experiment.
- Dependent variables, or outputs from the system, where we can classify the temperature map. Indeed, as it happens in meteorology, temperatures are the final output of the system, which depends on the heat balance applied to the system.

Temperature can be measured by different types of instruments, such as thermocouples and thermistors, with a cost that is a very small fraction of the investment needed to build a test hall. This is an important cost reference, although some proposals have been made to reduce the cost of an official test station, including a study of the difference between prototypes and units of industrial series [30,31].

The ATP method does not take into account the coherent relation between power and temperature, just the contrary. It fixes a temperature level for making the test, which corresponds to 25 °C of difference between the inner (inside the box) and outer air (test hall). So, the power level has to be moved until it converges to that point. Of course, this method can be used (and it is used) thanks to control and feedback, but it goes against the physics of the problem, as described by Fourier in relation to the radiation budget between the Sun and the Earth, and it makes tests longer than needed (most of the customers of the test stations hate leaving the unit for more than 12 h for the test). Besides that, ATP only requires one experimental evidence, centered in the 25 °C as already said. Does this level have any special significance? Not at all. Are the rest of the possibilities of lower importance? Not at all. It was just chosen to have the same point for everyone, but this point does not add any special value to the experiment.

Another important reference is supplied by the time needed for the test because it gives an indication of the price of the test. The ATP Treaty requires more than 12 h in steady state and a previous phase of 6 h of preparation. So, ATP tests for determining K require at least one day, which conveys a sizeable loss of money for the owner of the body.

Electric active power heating the inside of the box is required by the Treaty to have an uncertainty better than 0.5%. Additionally, temperatures must be measured with an accuracy better than 0.1 °C, which represents a relative error of approximately 0.4% for the prescribed temperature difference of 25 °C. A further source of instrumental uncertainty arises from the determination of the representative surface *S*, whose geometric measurement typically contributes an additional ≈2%. When these three independent uncertainties are propagated according to the ATP formulation of $K=\frac{Q}{S\cdot (T_{i}-Te)}$, the resulting instrumental uncertainty is approximately 2–2.3%. However, this estimate only accounts for the inherent uncertainties of the measuring instruments; it does not represent the actual uncertainty of the physical problem, which is dominated by the behavior of the heat-transfer mechanisms involved.

In contrast, the optimized protocol proposed in this work significantly reduces the effective uncertainty in the determination of K. First, the method acquires a large number of independent stabilized measurements of Q and T, which provides statistical significance and reduces the standard uncertainty by a factor $1/\sqrt{N}$. In the experiments shown in Table 3 and Table 4, 10–20 valid samples are routinely obtained, yielding a relative uncertainty of approximately 0.7–1.0%. Second, the shortened test duration strongly mitigates physical effects that can bias the measurement, such as air leakage driven by the chimney effect or latent heat losses associated with water evaporation from the inner surfaces. These phenomena tend to become more pronounced during long ATP tests, but are largely suppressed in the proposed protocol. As a result, the revised procedure not only reduces the testing time but also improves the accuracy and physical representativeness of the measured K value.

The problem can be featured as a heat conduction case surrounded by convection streams. The total thermal resistance from inner air (recirculated inside the box or tank) and outer air (recirculated in the test hall) is the addition of the conduction resistance (which accounts for 95 % of the total, in general) plus the convection resistance of both boundary layers. Current ATP test specifications do not prescribe any insight into the thermal structure of the problem, and it only works with air temperatures inside and outside, but the case is basically governed by the conduction problem.

The coefficients of the heat equations represent the physical characteristics of the materials used in the tested body. Three properties appear originally in the general equation:

- Thermal conduction, θ.
- Density, ρ.
- Specific heat, *C*.

All three coefficients are lumped in the thermal diffusivity, α, as already seen. This is the only acting parameter in the time-dependent heat conduction equation, and it is the physical variable controlling the length of the transients.

Transient time spans are expressed in terms of $F_{o}$ number, and most of the transients finish before $F_{o}$ reaches 0.5 (0.7 can be considered a very upper limit for that). A diffusivity of 10^−6^ m^2^/s would lead to transients of 4.000 s in walls 10 cm thick. Some wall materials have smaller diffusivity, which implies longer transient times (a few hours).

Once the transient is finished, the temperature distribution in the steady state gives the value of coefficient K (in fact, the test only gives the heating power and the temperature difference).

A review of the stability of the physical properties of the insulators points out that their values remain fairly invariant across the temperature domain usually found in ATP, which can cover from −20 °C to +50 °C. In general, all materials have very uniform values of thermal properties until a phase change arrives. In that case, the full integrity of the wall could not be preserved, and the box or tank would remain useless.

In fact, disturbances in the thermal problem can stem from other roots, particularly the presence of water inside of the unit walls, and air leaks from inside to outside. In both cases, they represent actual heat sinks that disturb the problem, either for a while (water evaporation) or permanently (chimney effect).

Liquid water can be found (in small amounts) inside the floor of the box, usually as a byproduct of cleaning it. When the heat arrives inside the structure of the floor, liquid water enters an evaporation process that needs 2.4 kJ per gram of water, which is a non-negligible number. Note that a box qualified as IR will have a coefficient not higher than 0.4 kJ/m^2^°C, which means 10 kW/m^2^ if the difference between temperatures is fixed at 25 °C. If the floor has a charge of 10 g/m^2^ of water, the thermal energy required to evaporate it would be close to 24 kJ/m^2^. The disturbances created by evaporation will affect the experiment for a much longer time (between 100 and 1000 times the time equivalent of full dedication to evaporate, but the evaporation process will induce a fake increase in thermal resistance that will be 100 times lower, or even 1000). Water will not be a real problem in most cases, although some floors have wooden platforms where the amount of retained water can be much higher than 10 g/m^2^. If this is so, the experiment will need an extra initial stage to get rid of that water.

The second cause of thermal resistance disturbances is air leaks. This is in turn related to a topic not well treated in the ATP Agreement, where some emphasis is put on the hermetic condition of the box or the tank. The text is written under the assumption that air leaks do not occur in ATP boxes or tanks, which is not true, as already analyzed in previous paragraphs.

In ATP tests (with internal heating), air can leak from inside due to chimney effects, and this effect implies a very bad qualification of the unit, that should be repaired for remaining active as an ATP unit. The existence of this effect in a given unit will be revealed by a test as the one shown in Figure 16 and discussed in Figure 17. Indeed, the method of making successive variations of the power level heating the inside of the unit is proposed in this paper as the general protocol for running isothermal tests. The new protocol would convey a strong reduction in the time span of each phase (which will have a constant heating power level). Three phases are at least recommended for this purpose, with the following additional prescriptions (which are not totally stiff; they must be taken as recommendations):

- Air of the test hall has to be kept constant all the time, within a range between 5 °C and 12 °C.
- Heating power will have to be chosen for the first phase so that the inner air is 25 °C above the outer air, presuming a K coefficient just in the limit of the class intended for that unit (0.4 W/m^2^ °C for IR and 0.7 W/m^2^ °C for IN).
- The heating power for next phases (at least two) could be chosen by the test officer for having information covering a sizeable fraction of the domain represented by a range of the inner air temperature between 25 °C and 40 °C.

There is a final point to be dealt with in this analysis, and it is the experimental verification that no unusual phenomena disturb a test. This produces abnormal facts from the thermal coefficients. If such alterations in the coefficients really happen, the evolution of the temperature along the first transient will show a disturbance. For instance, if the door of the box is open at a given moment in a test, the sudden change in the inner air temperature will point out that something abnormal has happened.

The concept of well-behaved response functions is of major importance to guarantee the stability of the coefficients governing a physical system. This is related to linear and non-linear systems. Linearity implies proportionality between causes and effects, while non-linear systems do not follow that principle, and a small change in one parameter causes enormous effects in some dependent variables.

In our case, according to what was already introduced, the independent variable is the heating power, and the dependent variables are the set of relevant temperatures characterizing the system. In a mere ATP scheme, there are only two temperatures for such a task: average temperatures of the inner and outer air. For a better characterization of the system and for the sake of having a more complete analysis of the behavior of the response functions, we have included in our experiments the surface temperature of each face of each wall of the box, which are 12 additional response functions.

Although the inner and outer air temperatures are taken as representative values, it should be noted that the wall surfaces are not strictly isothermal either. They exhibit slight spatial variations due to differences in local convection coefficients and geometric features of the container.

**Figure 19:**
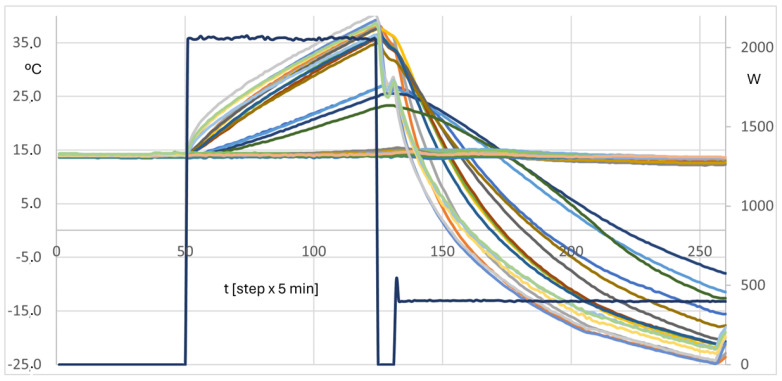
Temperature evolutions for different points in an ATP box, internally heated at a constant power of 2 kW from step 50 to step 129 (each step is 5 min). Temperatures are read in the left-hand-side axis. Heating power is the black line. The experiment does not belong to an ATP test. It is aimed at sweeping the whole range of temperatures.

**Figure 20:**
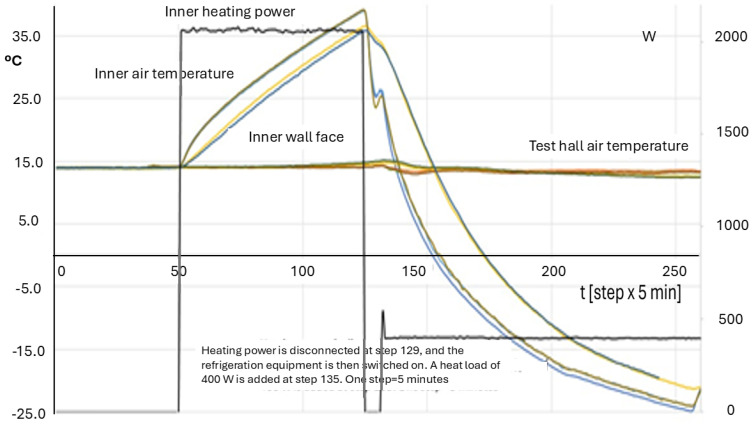
Additional information to Figure 19. The temperature in the test hall is slightly below 15 °C for most of the time.

Figure 19 and Figure 20 show the temperature evolution in a test of an ATP box along the first phase of heating. There are many functions, representing corresponding temperatures. The most important conclusion is that all functions can be classified as well-behaved because they have monotonous evolutions, which are coherent with the Physics of the problem at this stage. Recorded temperatures do not show jumps, oscillations or other disturbances. They only reflect changes in the heating power, which is the independent or guiding variable.

Figure 19 shows all the recorded temperatures. All curves of the same family are practically the same. The behavior is really good, both in the heating phase and in the cooling one. Heating power is kept constant at 2.06 kW from steps 50 through 129, and the refrigeration equipment is then switched on. A heat load of 400 W is added at step 135. One step lasts for 5 min. In Figure 20, some selected information is depicted, concerning the inner air and the inner surface temperatures, in the lateral panel of the driver side. In this case, the refrigeration equipment power is stronger than the heating power, and the cooling transient happens in shorter times. In the switch from heating to cooling, the air and the surface temperatures change the relative position. The air temperature shows a small oscillation when the heat load is added in the cooling stage, as it should be.

The test protocol can specify more details, such as the number of places to register the temperature of the test hall, and it will have a criterion for stating that the situation of the experiment is in a steady state, and representative measurements can be made. It will be advisable to compute and register the ratio W/ΔT, or directly K.

All experimental boundary conditions reported in this section strictly comply with the requirements of the ATP standard. In particular, the air velocity in the test hall was maintained within the prescribed range of $\left(1.58\pm 0.30\right)\;ms^{-1}$, and the external air temperature was controlled within (8.0±0.5)°C. These values fall entirely within the ATP acceptance intervals specified for isothermal testing and were continuously monitored throughout the experiments.

A steady state has to comply with the following specifications, along 1 h at least:

- The average power level (W) is defined as the total active energy consumed by all electric appliances producing heat inside the equipment along the whole duration of the steady state, divided by the total time span of the steady state. Every 5 min, a stepwise power level is calculated as the active energy consumed during that period, divided by 300 s. For the steady state to be acceptable, all stepwise power levels must not differ more than 1 % from the average power level. If this is so, the power level recognized for this steady state is the average power level.
- In an isothermal test, temperatures will be measured and recorded every minute, at the following points of the equipment or nearby:
  - Twelve points specified by the original ATP methodology, to measure the temperature of the air outside the equipment, with a circulation speed between 1 and 2 m/s. The mean value of the 12 recordings will be considered the relevant temperature of the outer air. This temperature must be between 5 °C and 10 °C for a steady state to be accepted as such.
  - Twelve points specified by the original ATP methodology, to measure the temperature of the air inside the equipment, with a recirculation flow between 40 and 70 times the internal volume, per hour. The mean value of the 12 recordings will be considered the relevant temperature of the inner air. This temperature must be between 20 °C and 35 °C for a steady state to be accepted as such.
  - Three points on the internal faces of the walls of the equipment, using contact thermometers. One of these points must be in a lateral wall, another in the front or rear wall, and the third one either on the floor or the ceiling. Each of these contact temperatures will be registered on its own.
  - Three points on the external faces of the walls of the equipment, using contact thermometers. One of these points must be in a lateral wall, another in the front or rear wall, and the third one either on the floor or the ceiling. Each of these contact temperatures will be registered by its own
- For a steady state to be accepted as such, the relevant temperature of the inner air, the relevant temperature of the outer air and every contact temperature on the internal and external faces of the walls must comply individually with the following: there will be a set of stepwise mean values calculated with the five measurements of 5 min, consecutively, and there will be an average value of the whole steady state. For each of the eight temperatures identified in this paragraph, all stepwise mean values will differ less than ±0.5 °C from the steady-state average value.
- Once the former requirements on temperatures and active heating power are verified, the steady state will be characterized by a power level W, which will be the formerly defined average power level, and by a difference in air temperatures, ΔT, which will be the difference between the relevant temperature of the inner air minus the relevant temperature of the outer air. The steady state will also be characterized by its specific thermal ratio, which is given by W/ΔT (for algebraic purposes, this ratio will be represented by the letter Z).
- Once a steady state has been accepted as such, the test will be sent into a transient devised to produce a new steady state, heated by a power level that must be either higher than 115 % or lower than 85 % of the value of the previous steady state. The new steady state will have to comply with the same requirements formerly given.
- The specific thermal ratios of the first and the second steady states, Z1 and Z2, respectively, will be used for checking the validity of the test. If the absolute value of the difference (Z1 − Z2) is smaller than 2.8 % of the ratio mean value, (Z1 + Z2)/2, the test is accepted and it is characterized by this mean value. Any other parameter of the test will be the mean value of the corresponding parameters of both states (2.8 % is a measure of the accuracy sought in the test, and could be changed to another value).
- If the test is not valid according to the former criterion, a third steady state must be obtained, and the full set of steady states will be treated as a sampling of the features of the equipment; the mean value µ and the standard deviation of the set, σ, will be calculated by
  (41) $$\mu =\sum_{1}^{3}Z_{i}/3$$
  (42) $$\sigma =\sqrt{\sum_{1}^{3}{\left(Z_{i}-\mu \right)}^{2}/2}$$
- The test will be accepted when
  (43) 2·σ≤0.04·μ
- If n steady states have been obtained in a test, the corresponding values of μ and σ will be
  (44) $$\mu =\sum_{1}^{n}Z_{i}/n$$
  (45) $$\sigma =\sqrt{\sum_{1}^{n}{\left(Z_{i}-\mu \right)}^{2}/\left(n-1\right)}$$

The former protocol is just an example of how to generate and manage statistical significance. Of course, the number of registering points outside, inside and on the walls is something to be discussed in an open seminar or technical commission.

One important question must also be included in this protocol: what to do with units that show the effects of chimneys, i.e., units that have a K coefficient that increases as the heating power does. The answer seems to be clear: reject it as an ATP unit and send it to be repaired (if the owner wants to keep it in the business).

A last contribution of this work deals with the interpretation of the evolution of the measurements over time, and how to assess if the test is going in the right direction. First of all, according to the previous paragraphs, the right direction means that the test is going towards a steady state. This is clearly defined by a constant value in all relevant variables, namely

- Air temperature in the test hall (which actually is a boundary condition);
- Heating power, which is the input variable;
- Average temperature of the air inside the unit, which is the output variable.

**Figure 21:**
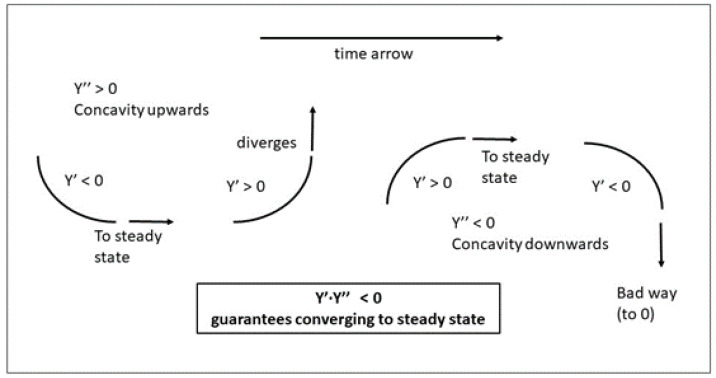
The evolution of the system towards a steady state is guaranteed by the mathematical fact that the product of the time derivative of the output variable times the second-order derivative of the same variable is negative, and decreasing in absolute value (until arriving at 0).

**Figure 22:**
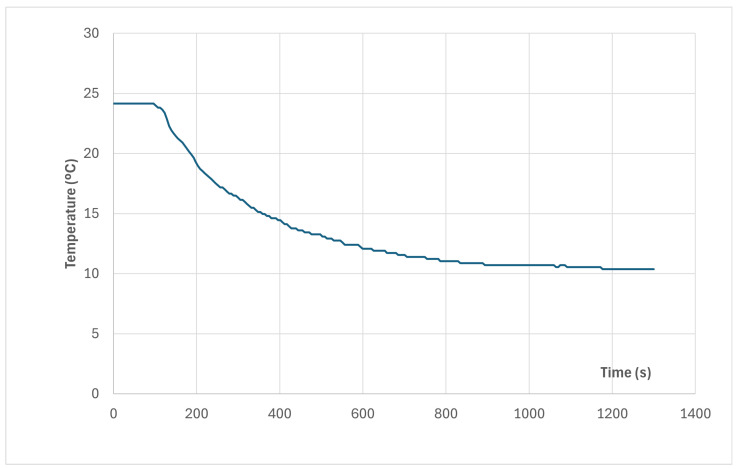
Temperature evolution of a bucket of liquid water where some amount of ice is added.

**Figure 23:**
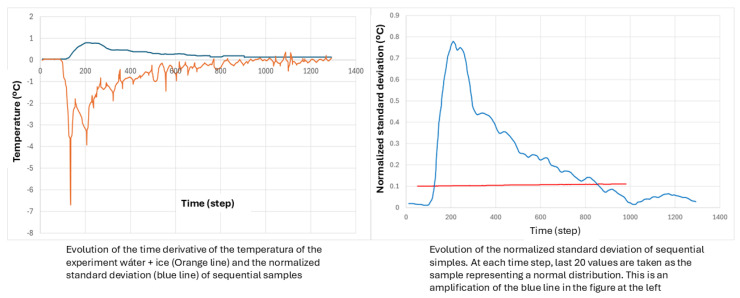
On the left-hand side, the time derivative of the evolution given in the previous figure is presented (orange line), as well as the standard deviation (divided by the mean value, and multiplied by 10, to represent it). On the right-hand side, an amplification of the standard deviation is shown.

The protocol should include a surveillance of the evolution of the test, to avoid growing problems that can destroy the test. As shown in Figure 21, this can be achieved by calculating and checking the first-order time derivative and the second-order time derivative of the output variable; and to check that its product is negative and decreasing in absolute value. This check can be performed automatically; but it must be noted that first- and second-order derivatives of an experimental recording can present very strong oscillation, because they are calculated as the difference of two very close numbers, and the raw application of this criterion can induce errors of judgement. One possibility is to obtain a smooth curve by fitting the experimental results and use this curve as the true performance of the test, and another possibility, which is original as far as the authors know, is to treat the time-dependent evolution of a given variable as a sample of a normal distribution and to qualify the evolution as a steady state when the normalized standard deviation is smaller than the uncertainty associated with this variable. Information given in Figure 22 and Figure 23 will be helpful to explain this approach.

The evolution of the temperature is treated in this method as a moveable sampling, in such a way that, at a given time step, a number of registers are considered as a sample. In this experiment, the recording time step was 5 s, and the sample included the last 20 time steps. The experiment is very simple so the physics of the problem gives good support to the statistical representation. The experiment was aimed at measuring the effects of ice melting in a bucket of water (2 kg of ice, initially at −19 °C, in 15 L of water, initially at 24 °C; final temperature 10.5 °C). Melting is fully completed in 20 min (1200 s). Before pouring the ice, the standard deviation was almost zero. After full melting, with the new thermal equilibrium, the standard deviation also was very close to zero. A red line at an ordinate 0.1 shows in the figure on the right the level of uncertainty assumed in this experiment, which really corresponds to 0.01 of the mean, and this was 0.24 °C of standard deviation before the experiment, and 0.1 °C after the experiment (in the new thermal equilibrium). It must be added that the temperature was measured with several thermocouples, but only the mean value was used to interpret the results. In this case, the transient could be considered over in 1200 s (20 min). Similarly, this method could be used in ATP official tests to decide if a new steady state has been reached (a new thermal equilibrium).

These results show that natural convection and leakage effects increase with test duration, providing a physical justification for the accelerated protocol proposed in this work, which minimizes the influence of these phenomena by shortening the stabilization and measurement phases.

## 4. Conclusions and Future Work

Technical devices for the transportation of perishable foodstuff are bound to undergo important changes because of environmental requirements and energy savings. Although some regulations, such as the one by the European Union on fluorinated gases [32], do not yet include this type of transportation in their restrictions, it is obvious that a technology update can be achieved and must be achieved [33,34] because there are ways and means to do it.

Some reviews were undergone (already in this Century) to classify potential deployments of these technologies [35,36,37] but companies cannot go simply towards their own objective, because a new text of the ATP Agreement could totally modify the type of units that could be sold. Standards and their tests have to be updated before updating the industry itself. Moreover, it is worth recalling that refrigeration systems have demonstrated very good performance in the last 20 years, and air conditioning has become a complete household device. If ATP devices have to evolve, official tests to measure their performance will have to change accordingly. In this context, the isothermal test needs to be redressed, because it has a very bad definition, namely, the test must be run for a lot of hours (about 18 at least) but with a very low frequency (once every 15 min; reduced to 5 min in the last version of July 2021).

This situation corresponds to a wrong estimate of the duration of transients, and a worse estimate of the time span needed to guarantee that a thermal system is in a steady state. A steady state is kept as such once the previous transient has disappeared, provided all boundary conditions are maintained as they were, and the physical systems are not perturbed by any external agent. Both conditions can easily be verified by the succession of measurements, namely, the air temperature in the test hall, the heating power inside the unit and the air temperature inside the unit as well. It can be complemented by registering the temperature of the surfaces of the wall in a number of points for improving the knowledge of the system.

In all relevant temperatures registered, the evolution of the first- and second-order time derivatives can be used to make sure that the system is evolving towards a steady state (T′·T″ < 0) or it already is in it, which can be defined by T′ = 0 (which is a criterion difficult to implement, because of the oscillating character of the temperature).

A new methodology is proposed for the classification of ATP items, conceived as a reformulation of the classical procedure rather than as a complete replacement. The new approach preserves all the fundamental elements of the original ATP model, ensuring full compatibility with existing databases and previously certified results. In this sense, historical ATP data remain directly comparable with those obtained using the optimized protocol.

The preparation of the test follows the same initial steps as in the standard method. The ATP item is installed under identical conditions, and the same physical variables are monitored throughout the experiment, namely the internal and external air temperatures and the electrical power supplied by the heating system. In addition to the instrumentation required by the current ATP procedure, six supplementary temperature sensors are installed at the locations specified in Section 4, in order to improve spatial representativeness and allow a more robust assessment of thermal stabilization.

A clear and operational criterion is defined to determine when the system has reached a steady state. This condition is associated with the convergence of the average internal air temperature to a stable value *T* and with the establishment of a constant electrical heating power *W* within prescribed tolerance limits. Once this stabilized regime is achieved, the test proceeds by deliberately perturbing the system: the electrical power is either increased to more than 115% of its stabilized value or reduced below 85%, thereby forcing the system to evolve toward a new steady state.

The experiment is continued until this second stabilized regime is reached. At that point, the values of *T* and *W* corresponding to both steady states are compared. If the consistency criteria defined in the previous section of the paper are satisfied, the global heat-transfer coefficient *K* is determined accordingly. If the criteria are not met, an additional power step is applied and the procedure is repeated until convergence is obtained. In practice, experimental results show that two power steps are generally sufficient to achieve reliable convergence.

Compared with the classical ATP method, which typically requires no less than 20 h to complete a single test, the optimized protocol significantly reduces the overall testing time. Using the proposed approach, a complete test can be performed in less than 10 h. This reduction makes it possible to carry out up to two ATP tests per day, effectively doubling the testing capacity of a certification facility without compromising measurement reliability or compatibility with existing ATP classifications.

To illustrate the quantitative impact of steady-state duration on the determination of the global heat-transfer coefficient, Table 5 reports the values of *K* obtained for three representative ATP items using different stabilization times. The table also includes the maximum deviation observed for each case, providing a direct measure of the sensitivity of *K* to test duration.

A more practical alternative is to use a statistical study, as the one presented in the previous section. Moreover, both analytical theories and experimental results have been used to guarantee that high-quality tests can be performed with new prescriptions fully backed by theory and practice.

This study has several limitations that should be acknowledged. First, the proposed accelerated protocol has been validated on a representative but limited set of ATP containers, and its quantitative performance may vary for units with atypical wall compositions, geometries or extreme humidity conditions. Second, the analysis focuses on isothermal tests with internal electric heating; refrigerated units operating with mechanical refrigeration systems may require additional verification steps to account for compressor cycling and dynamic control strategies. Third, although the method improves both accuracy and testing time, it does not replace the legal status of the current ATP procedure, which remains defined by international agreement.

Future work should extend the experimental database to other container types and climatic conditions, investigate the robustness of the protocol under different operational constraints and explore the integration of real-time monitoring data into the testing methodology. It will have to follow the tracks already opened in this paper so that simpler and shorter tests can be performed for qualifying ATP equipment and units. There is a certain lack of thermal coherence [38] in the current ATP specifications, and there are several lines of research [36] pointing out that the time for updating them has already arrived for a better service in perishable foodstuff transportation.

## Figures and Tables

**Table 1:** Amplitudes of the first modes of a uniform function.

Mode *n*=	0	1	2	3	4	5
** $T_{n}$ **	1.2739	−0.4246	0.2548	−0.1820	0.1415	−0.1158

**Table 2:** Amplitudes of the first modes of a parabola.

Mode *n*=	0	1	2	3	4	5
** $T_{n}$ **	1.0320	−0.0382	0.0083	−0.0030	0.0014	−0.0008

**Table 3:** For case reported in Figure 14: average values, standard deviation and its normalized values for heating power and temperature for different ranges within the already defined first stage.

Range	W	SD (W)	Normalized	T (°C)	SD (T)	Normalized
500–1000	545	9.6	0.0175	32	0.031	0.001
500–700	544	9.9	0.0180	32	0.033	0.001
500–600	545	11.2	0.0200	32	0.034	0.001
500–550	543	11	0.0200	32	0.035	0.001
500–525	539	7.9	0.0150	32	0.035	0.001

**Table 4:** For case reported in Figure 14: average values, standard deviation and its normalized values for heating power and temperature for the time ranges of each stage.

Range	W	SD (W)	Normalized	T (°C)	SD (T)	Normalized
500–1000	545	9.6	0.0175	32	0.031	0.001
1200–1600	581	9.8	0.0165	32.5	0.031	0.001
1700–2300	621	10.7	0.0172	33	0.032	0.001
2600–2800	507	8.2	0.0160	31	0.033	0.001

**Table 5:** Stability of the global heat-transfer coefficient *K* as a function of steady-state duration in ATP tests.

ATP Test	18 h	12 h	9 h	6 h	Max-Min Deviation
(7015) k=	0.6791	0.6795	0.6780	0.6714	0.0081
(7075) k=	0.3828	0.3814	0.3793	0.3706	0.0122
(7038) k=	0.4022	0.4008	0.4041	0.4039	0.0033

## Data Availability

Data is unavailable due to privacy.

## Abbreviations

The following abbreviations are used in this manuscript:

ATP	Agreement on the International Carriage of Perishable
	Foodstuffs and on the Special Equipment to Be Used for Such Carriage
IN	Normal insulation class
IR	Reinforced insulation class
UNECE	United Nations Economic Commission for Europe
Latin symbols	
*A*	Area, m^2^
*C*	Specific heat, kJ kg^−1^K^−1^
*D*	Hydraulic diameter, m
*H*	Wall thickness, m
*K*	Global heat-transfer coefficient, Wm^−2^K^−1^
*L*	Characteristic length (leakage path), m
*Q*	Heating power, W
*S*	Representative surface of the unit, m^2^
$S_{\mathrm{avg}}$	Effective conduction surface, m^2^
*T*	Temperature, °C or K
*t*	Time, s
*v*	Leakage speed, ms^−1^
*Z*	Specific thermal ratio, WK^−1^
Greek symbols	
α	Thermal diffusivity, m^2^s^−1^
θ	Thermal conductivity (as used in the text), Wm^−1^K^−1^
ρ	Density, kgm^−3^
Dimensionless numbers	
Fo	Fourier number, $\mathrm{Fo}=\alpha t/H^{2}$
Re	Reynolds number
